# BMP- and neuropilin 1-mediated motor axon navigation relies on spastin alternative translation

**DOI:** 10.1242/dev.162701

**Published:** 2018-09-12

**Authors:** Nicolas Jardin, François Giudicelli, Daniel Ten Martín, Anaïs Vitrac, Stéphanie De Gois, Rachel Allison, Corinne Houart, Evan Reid, Jamilé Hazan, Coralie Fassier

**Affiliations:** 1 Sorbonne Universités, UPMC Université Paris 06, INSERM, CNRS, Neuroscience Paris Seine - Institut de Biologie Paris-Seine (NPS-IBPS), 75005 Paris, France; 2 Sorbonne Universités, UPMC Université Paris 06, CNRS, INSERM, Biologie du Développement Paris Seine - Institut de Biologie Paris-Seine (LBD-IBPS), 75005 Paris, France; 3 Cambridge Institute for Medical Research, University of Cambridge, Cambridge CB2 OXY, UK; 4 Medical Research Council Centre for Developmental Neurobiology, King's College London, London SE1 1UL, UK

**Keywords:** Axon navigation, BMP signalling, Hereditary spastic paraplegia, Neuropilin 1, Spastin, Zebrafish

## Abstract

Functional analyses of genes responsible for neurodegenerative disorders have unveiled crucial links between neurodegenerative processes and key developmental signalling pathways. Mutations in *SPG4*-encoding spastin cause hereditary spastic paraplegia (HSP). Spastin is involved in diverse cellular processes that couple microtubule severing to membrane remodelling. Two main spastin isoforms are synthesised from alternative translational start sites (M1 and M87). However, their specific roles in neuronal development and homeostasis remain largely unknown. To selectively unravel their neuronal function, we blocked spastin synthesis from each initiation codon during zebrafish development and performed rescue analyses. The knockdown of each isoform led to different motor neuron and locomotion defects, which were not rescued by the selective expression of the other isoform. Notably, both morphant neuronal phenotypes were observed in a CRISPR/Cas9 *spastin* mutant. We next showed that M1 spastin, together with HSP proteins atlastin 1 and NIPA1, drives motor axon targeting by repressing BMP signalling, whereas M87 spastin acts downstream of neuropilin 1 to control motor neuron migration. Our data therefore suggest that defective BMP and neuropilin 1 signalling may contribute to the motor phenotype in a vertebrate model of spastin depletion.

## Abstract (precis)

**Summary:** Spastin main isoforms play crucial and specific roles in zebrafish motor circuit wiring and larval locomotion through their distinct regulation of the BMP and neuropilin 1 axon guidance pathways.

## INTRODUCTION

Various neurodegenerative disorders have been shown to result from the primary failure of selectively vulnerable neurons during embryonic and postnatal development of the nervous system. Indeed, proteins implicated in early- and late-onset neurodegeneration are massively expressed throughout embryogenesis and frequently impact on different steps of neural development, such as neuronal proliferation, differentiation and migration, axon targeting or synaptogenesis. Furthermore, these affected molecular bases underlying belated neurodegenerative processes are often embedded into key developmental signalling pathways in both invertebrate and vertebrate pathogenic models, such as the Notch (Levitan and Greenwald, 1995) and Wnt (Nishimura et al., 1999) pathways in Alzheimer's disease, Wnt signalling in Huntington disease (Dupont et al., 2012; Tourette et al., 2014) or the bone morphogenetic protein (BMP) pathway in motor neurodegeneration (Wang et al., 2007; Chang et al., 2008; Ratnaparkhi et al., 2008; Fassier et al., 2010; Nahm et al., 2013).

Hereditary spastic paraplegia (HSP) is a heterogeneous group of neurological disorders mainly characterised by progressive spasticity of the lower limbs due to the degeneration of the cortico-spinal tracts. HSP shows an extreme genetic heterogeneity with more than 84 spastic paraplegia gene (*SPG*) loci reported so far (Tesson et al., 2015). Mutations in the *SPG4* gene encoding the multifaceted microtubule-severing spastin cause the major form of autosomal dominant HSP (Hazan et al., 1999). More than 200 mutations have been described within *SPG4* and the vast majority of them are likely to act via haploinsufficiency (Fonknechten et al., 2000; Roll-Mecak and Vale, 2008; Riano et al., 2009). Spastin is essential for numerous cellular processes (Connell et al., 2009; Park et al., 2010; Allison et al., 2013, 2017; Papadopoulos et al., 2015; Vietri et al., 2015) linking microtubule (MT) severing to membrane remodelling (Blackstone et al., 2011; Lumb et al., 2012). In developing neurons, spastin is required for axonal and synaptic growth (Sherwood et al., 2004; Trotta et al., 2004; Wood et al., 2006; Riano et al., 2009) as well as neurite branching or pruning (Yu et al., 2008; Brill et al., 2016), whereas in mature neurons it preserves axonal maintenance and transport homeostasis (Tarrade et al., 2006; Fassier et al., 2013; Denton et al., 2014; Havlicek et al., 2014). Notably, alternative translation of mammalian *SPG4* transcript directs the synthesis of two main spastin isoforms (M1 and M87 isoforms; Claudiani et al., 2005), which show different structural domains, tissue and subcellular distribution, as well as binding partners (Evans et al., 2006; Sanderson et al., 2006; Connell et al., 2009; Park et al., 2010; Solowska et al., 2010; Montenegro et al., 2012; Papadopoulos et al., 2015). However, although spastin function has been intensively investigated, most functional studies were performed in non-neuronal cell lines and failed to address the roles of these spastin isoforms *in vivo* and their respective contribution to HSP pathogenesis. A few arguments suggested a major involvement of the M1 isoform: it is enriched in the developing and adult spinal cord (Claudiani et al., 2005; Solowska et al., 2010), the overexpression of its pathogenic forms have toxic effects compared with that of M87 (Solowska et al., 2008, 2014; Leo et al., 2017), and it selectively binds to other known HSP proteins (Evans et al., 2006; Sanderson et al., 2006; Park et al., 2010; Montenegro et al., 2012), including the BMP inhibitor atlastin 1 (Fassier et al., 2010; Zhao and Hedera, 2013), which suggested a specific role for this long isoform in the BMP pathway (which is known to be dysregulated in different subtypes of HSP) (Wang et al., 2007; Tsang et al., 2009; Fassier et al., 2010; Renvoisé et al., 2012; Nahm et al., 2013; Song et al., 2013; Mao et al., 2015).

Here, loss-of-function and rescue analyses during zebrafish embryogenesis allowed us to reveal that spastin isoforms show non-overlapping key functions in spinal motor neuron development and larval locomotion, whereas their specific involvement in the BMP or the neuropilin 1 axon guidance pathway was unravelled.

## RESULTS

### Zebrafish *spastin* transcript drives the synthesis of two spastin isoforms with distinct subcellular distribution in developing motor neurons

**Fig. 1.**
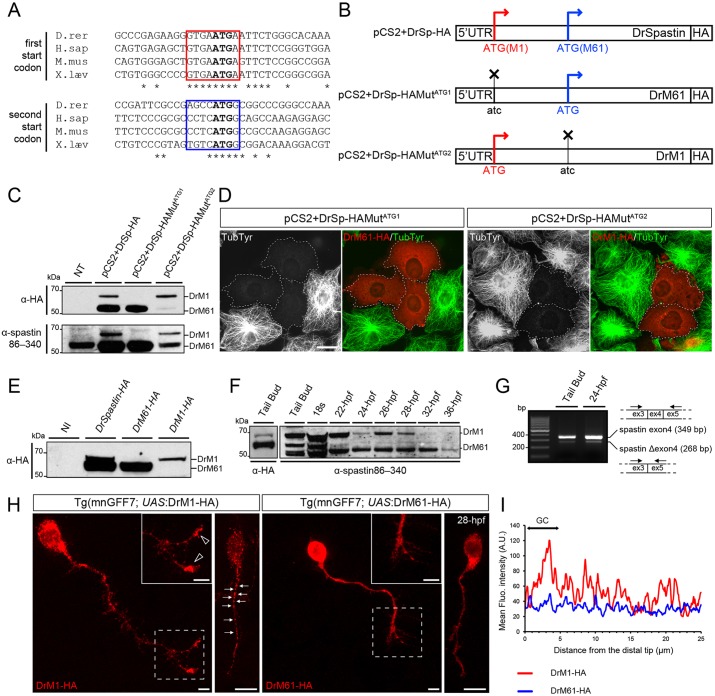
**Two main spastin isoforms are synthesized during zebrafish development through alternative use of two initiation codons.** (A) Alignment of spastin cDNA sequences surrounding the first and second ATG (bold) from different vertebrate species. Kozak sequences surrounding the first and second ATG are boxed in red and blue, respectively. Asterisks indicate conserved nucleotides. (B) Schematic representation of the different spastin constructs used in the present study. Crosses indicate mutated ATG. (C-E) Zebrafish *spastin* transcript drives the synthesis of two main spastin isoforms through alternative translation start sites. (C) Western blot analysis of spastin expression in COS-7 cells transfected or not (not transfected, NT) with the different spastin constructs (see B) using HA and spastin86-340 antibodies. (D) Both zebrafish spastin isoforms display a microtubule-severing activity *in vitro*. Immunolabelling of spastin and microtubules in COS-7 cells overexpressing DrM1 or DrM61-HA using HA (red) and tyrosinated tubulin (green) antibodies. Dotted lines surround transfected cells. Both overexpressed DrM1 and DrM61 dismantle the microtubule network. Scale bar: 20 µm. (E) Western blot analysis of exogenous spastin expression from protein extracts of tailbud embryos injected or not with *in vitro* transcribed mRNAs from the different spastin constructs using HA antibody. (F) The expression ratio between spastin isoforms varies during zebrafish development. Western blot analysis of spastin main isoforms during zebrafish development, using spastin86-340 antibody. Protein extracts from tailbud embryos injected with *spastin-HA* mRNA and revealed using an HA antibody, were used as DrM1 and DrM61 benchmark. (G) Spastin exon 4 is alternatively spliced during zebrafish development. RT-PCR analysis of spastin transcripts at both the tailbud stage and 24 hpf using primers flanking the exon 4 of the zebrafish *spastin* gene. (H,I) Distinct subcellular distribution of spastin main isoforms in spinal motor neurons *in vivo*. (H) Immunolabelling of 28 hpf Tg(mnGFF7;*UAS*:DrM1-HA) and Tg(mnGFF7;*UAS*:DrM61-HA) transgenic embryos with an HA antibody. Arrows indicate the punctate distribution of DrM1-spastin along the axon shaft while empty arrowheads indicate its specific enrichment in spinal motor growth cones. Scale bars: 10 µm. (I) Mean fluorescence intensity profile of DrM1 and DrM61 staining along the distal part of SMN axons (*n*=6 and *n*=8, respectively).

All published *spastin* knockdown experiments in the zebrafish have been performed with antisense morpholino oligonucleotides targeted against the first ATG (Wood et al., 2006; Butler et al., 2010; Allison et al., 2013). Yet the presence of a second in-frame ATG (encoding methionine 61 and corresponding to human M87) in the teleost sequence has been reported (Wood et al., 2006; Fig. 1A). To assess whether alternative translation of zebrafish *spastin* transcript drives the synthesis of two main isoforms as in mammals, we cloned the HA-tagged version of zebrafish *spastin* cDNA, including the 5′ untranslated region (UTR) that has been shown to influence the expression ratio of the two isoforms (Claudiani et al., 2005), in an expression vector (pCS2+DrSp-HA). We also performed targeted mutagenesis of each ATG (pCS2+DrSp-HAMut^*ATG1*^; pCS2+DrSp-HAMut^*ATG2*^; Fig. 1B) and analysed the expression of wild-type and Mut^ATG^ Spastin-HA constructs in transfected COS-7 cells. Western blot analysis of total protein lysates from cells transfected with these constructs using an HA antibody revealed that these two ATGs are functional and drive the synthesis of two zebrafish spastin isoforms (Fig. 1C) of 62 kDa (DrM1) and 55 kDa (DrM61), both showing a microtubule-severing activity *in vitro* (Fig. 1D). The expression ratio of these two spastin isoforms was conserved between mammals and zebrafish, with the long isoform showing a far weaker expression, possibly owing to the presence of a poor, but highly conserved, Kozak sequence surrounding the first ATG (Fig. 1A,C). *In vivo*, similar results were obtained from zebrafish embryos injected with wild-type (*DrSpastin-HA*) or mutated (*DrM61-HA* and *DrM1-HA*) *spastin* mRNAs (Fig. 1E), which indicated that the synthesis of zebrafish spastin occurred through alternative use of the two initiation codons. As anti-human spastin86-340 antibody (Connell et al., 2009) recognised overexpressed DrM1 and DrM61 in COS-7 cells (Fig. 1C), we used this antibody to assess the endogenous expression of these two isoforms during zebrafish development. Both isoforms were expressed at all embryonic stages from 12 to 36 h post-fertilisation (hpf). An additional smaller band of 52 kDa was also detected at all developmental stages (Fig. 1F), most likely corresponding to a splice variant of the short isoform. Indeed, we observed that: (1) *spastin* exon 4 could be alternatively spliced at these embryonic stages, as previously described in mammals and nematodes (Fig. 1G; Svenson et al., 2001; Matsushita-Ishiodori et al., 2007); and (2) this shorter variant was not produced from *in vitro* transcribed *DrSpastin-HA* mRNA, but (3) was equally immunolabelled by the S51 spastin antibody (data not shown; Errico et al., 2004). This splice variant was the major short isoform from the 18-somite stage onwards (Fig. 1F). Moreover, the expression ratio between the long and the short isoforms significantly varied throughout early stages of development, especially at key time points of spinal motor neuron development (between 18 somites and 32 hpf). This ratio was mainly influenced by the marked and reproducible variations in DrM1 expression, which suggested a tight regulation of the isoform expression during zebrafish motor neuron development (Fig. 1F). To investigate their subcellular distribution and overcome the lack of specific antibody against the long isoform, we generated two transgenic lines expressing HA-tagged versions of DrM1 or DrM61 under the control of a *UAS* promoter. By crossing these transgenic fish with the Tg(mnGFF7) fish (Asakawa et al., 2008, 2013) that express the transactivator GAL4 in spinal motor neurons (SMN), we observed that each isoform displayed distinct subcellular distribution in these neurons (Fig. 1H). DrM1 showed a punctate distribution within the soma and all along the axon shaft (arrows, Fig. 1H) with a clear enrichment in the growth cone (arrowheads, Fig. 1H,I and Fig. S1), whereas DrM61 was diffusely expressed in the whole neuron and mainly in the cytoplasm.

### The knockdown of each spastin isoform differentially affects zebrafish morphology and locomotion behaviour

**Fig. 2.**
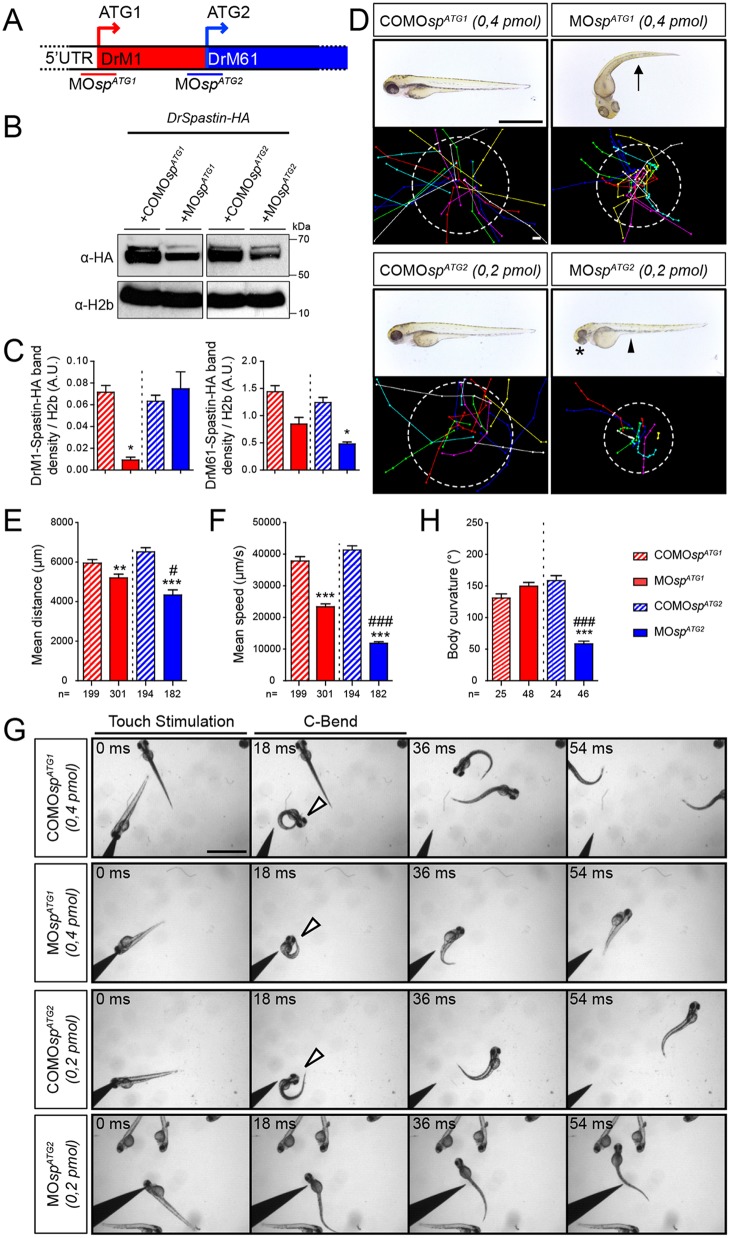
**The knockdown of each spastin isoform differentially alters larval locomotion.** (A) The knockdown of spastin main isoforms using a morpholino-based strategy. MO*sp^ATG1^* and MO*sp^ATG2^* morpholinos were designed to block spastin synthesis from the first (M1) or the second (M61) ATG. (B) Western blot analysis showing the efficiency of spastin isoform knockdown. Spastin expression was analysed in protein extracts from tailbud embryos injected with MO*sp^ATG1^*, MO*sp^ATG2^* or their corresponding control morpholinos using an HA antibody. H2b was used as a loading control. (C) Quantification of DrM1 and DrM61 band density normalised to H2b values (A.U., arbitrary units), from four independent experiments. *P≤0.05. Non-parametric Mann–Whitney test. Error bars are s.e.m. (D) Overall morphology (upper panels) and locomotor behaviour (bottom panels) of 72 hpf larvae injected with MO*sp^ATG1^* (*n*=301), MO*sp^ATG2^* (*n*=182) or control morpholinos (COMO*sp^ATG1^*, *n*=199 and COMO*sp^ATG2^*, *n*=194). Upper panels: Arrow indicates the curved-tail phenotype of ATG1 morphants. Asterisk and arrowhead show smaller eyes and yolk tube agenesis in ATG2 morphants. Bottom panels: Tracking analysis of 72 hpf control and morphant larvae in a touch-escape response test. Each line represents the trajectory of one larva after touch stimulation, while the dotted circle radius symbolizes the mean swimming distance covered by all larvae. Scale bars: 2 mm. (E) Quantification of the mean swimming distance. (F) Quantification of the mean swimming speed. (G,H) Touch-evoked startle response of 72 hpf COMO*sp^ATG1^* (*n*=25), COMO*sp^ATG2^* (*n*=24), MO*sp^ATG1^* (*n*=48) and MO*sp^ATG2^* (*n*=46) larvae. (G) Representative time series of control and *spastin* morphant larvae responding to a tactile stimulation. White arrowheads indicate the characteristic high body curvature (called C-bend) occurring after the touch stimulation. ATG2 morphants are unable to make the C-bend movement. Scale bar: 2 mm. (H) Maximal C-bend body curvature angle of the startle responses. (E,F,H) **P≤0.01, ***P≤0.001 versus control larvae; ^#^*P*≤0.05, ^###^*P*≤0.001 versus ATG1 morphants; Kruskal–Wallis ANOVA test with Dunn's post test. Error bars are s.e.m.

**Fig. 3.**
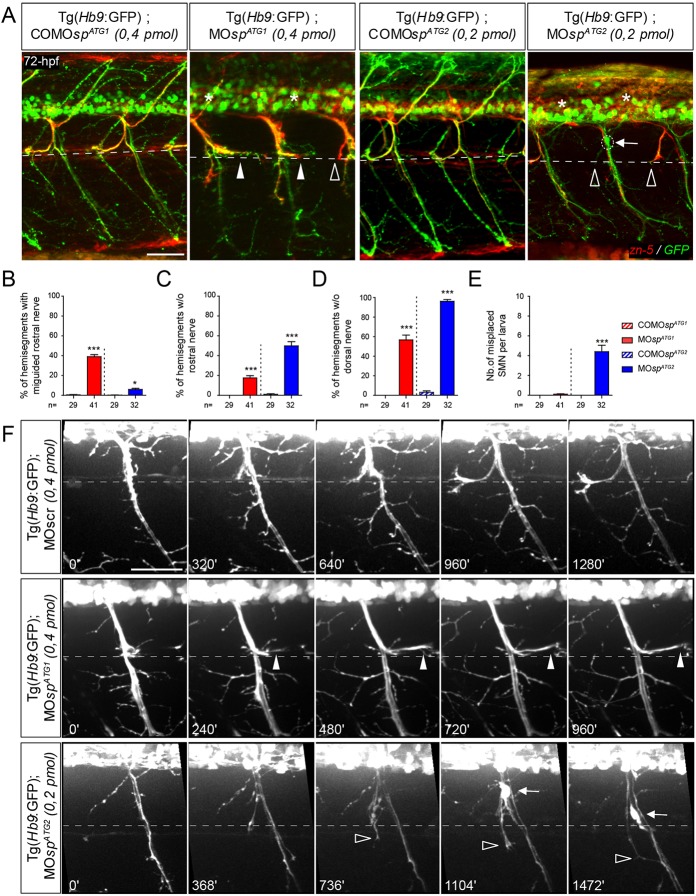
**ATG1 and ATG2 morphants exhibit different spinal motor neuron defects.** (A) Immunolabelling of secondary motor neurons (sMN) in 72 hpf COMO*sp^ATG1^* (*n*=29), COMO*sp^ATG2^* (*n*=29), MO*sp^ATG1^* (*n*=41) and MO*sp^ATG2^* (*n*=32) Tg(*Hb9*:GFP) transgenic larvae using zn-5 antibody. Lateral views of the trunk, anterior towards the left. Dotted lines indicate the horizontal myoseptum (i.e. axon guidance choice point). White arrowheads indicate misguided rostral nerves of ATG1 morphants while arrows indicate the ectopic sorting of sMN somata from the spinal cord of ATG2 morphants. Empty arrowheads and asterisks indicate missing rostral and dorsal nerves, respectively. Scale bar: 50 µm. (B-E) Quantification of sMN defects in 72 hpf control and *spastin* morphant larvae (24 spinal hemisegments were analysed per larva). *P≤0.05, ***P≤ 0.001; Kruskal–Wallis ANOVA test with Dunn's post test. The number (*n*) of larvae analysed per condition is indicated under the corresponding histogram bar. Error bars are s.e.m. (F) Distinctive navigational behaviour of ATG1 and ATG2 morphant sMN axons at the choice point. Representative stills of time-lapse recordings of sMN axon outgrowth monitored in 32 spinal hemisegments of 40-72 hpf Tg(*Hb9*:GFP) transgenic larvae injected with MO*sp^ATG1^*, MO*sp^ATG2^* or a control morpholino (MOscr). White arrowheads indicate the aberrant caudal turning of ATG1 morphant sMN rostral axons at the horizontal myoseptum (dotted line) while empty arrowheads indicate the erroneous ventral growth of ATG2 morphant sMN axons beyond this choice point. Arrows track the aberrant migration of sMN somata along motor tracts of ATG2 morphants. Scale bar: 50 µm.

To unravel the neuronal function of each spastin isoform, we took advantage of the morpholino-based knockdown strategy, which has the unique capacity to block protein synthesis from designated initiation codons without triggering the degradation of targeted transcripts, unlike RNA-interference approaches (Summerton, 1999). Morpholinos targeting the first (MO*sp^ATG1^*; Allison et al., 2013) or the second (MO*sp^ATG2^*) ATG were designed and injected into two-cell-stage embryos to downregulate DrM1 or DrM61 during zebrafish development (Fig. 2A). Mismatch (COMO*sp*^*ATG1*^ and COMO*sp*^*ATG2*^; Fig. 2) and scramble morpholinos (MOscr; Fig. 3) were used as controls. The knockdown efficiency of each spastin isoform was assessed by western blot analysis using protein extracts from embryos co-injected with MO*sp^ATG1^*, MO*sp^ATG2^* or the corresponding mismatch morpholinos and *DrSpastin-HA* mRNA, using an antibody against the HA tag (Fig. 2B). MO*sp^ATG2^* efficiently decreased DrM61 expression (61% reduction) compared with COMO*sp^ATG2^* without affecting DrM1 levels (Fig. 2B,C). In contrast, MO*sp^ATG1^* significantly and drastically reduced DrM1 expression (87% reduction) compared with COMO*sp^ATG1^* but not DrM61 levels (Fig. 2B,C). Both ATG1 and ATG2 morphants (i.e. embryos injected with MO*sp^ATG1^* or MO*sp^ATG2^*, respectively) failed to hatch from their chorion at larval stages but the two groups exhibited different morphological and locomotor phenotypes (Fig. 2D-F). The majority of ATG1 morphants showed a curved-tail phenotype (arrow, Fig. 2D), whereas all ATG2 morphants displayed smaller eyes (asterisk, Fig. 2D) and a yolk tube agenesis (arrowhead, Fig. 2D) compared with COMO*sp^ATG1^* and COMO*sp^ATG2^* larvae. Furthermore, a touch-escape response test at 72 hpf showed that both morphants displayed obvious locomotion defects, which were characterised by reduced swimming speed and covered distances compared with their related control larvae, and appeared to be more severe for ATG2 than for ATG1 morphants (Fig. 2D-F; Movies 1-4). In addition, ATG2 morphants showed atypical touch-evoked startle response compared with MO*sp^ATG1^*- or COMO*sp^ATG2^*-injected larvae. The zebrafish larval startle response triggered by a tactile stimulus may be fragmented into a series of stereotypic movement patterns, including a high-speed turn, called the C-bend, which occurs milliseconds after the stimulus and is required for escape (McClenahan et al., 2012). ATG2 morphants were unable to initiate a rapid C-bend movement in response to touch and reproducibly swam straight ahead over short distances compared with MO*sp^ATG1^*-, COMO*sp^ATG1^*- and COMO*sp^ATG2^*-injected larvae that showed a C-bend maximal body curvature to escape in the opposite direction from the stimulus (arrowheads, Fig. 2G,H). These distinct locomotion behaviours of ATG1 and ATG2 morphants suggested that the lack of DrM1 or DrM61 could differentially alter motor circuit formation.

### Spinal motor axon pathfinding is distinctively altered between the two spastin isoform morphants

Owing to morphant locomotion phenotypes, we analysed spinal motor neuron (SMN) development in ATG1 and ATG2 morphant larvae with a special focus on secondary motor neurons (sMN), which are key players in larval fast swimming and startle response behaviours. Immunolabelling of 72 hpf ATG1 or ATG2 morphant Tg(*Hb9*:GFP) transgenic larvae (Flanagan-Steet et al., 2005) revealed robust sMN migratory and/or axon pathfinding defects that were mainly specific to each morphant class. In ATG1 morphants, the rostral nerve appeared misguided in 39% of spinal hemisegments and abnormally grew caudally along the horizontal myoseptum (arrowhead, Fig. 3A,B), a phenotype rarely observed in COMO*sp^ATG1^*- (0.4%) or MO*sp^ATG2^*- (5.9%) injected larvae (Fig. 3A,B). In contrast, the rostral nerve of ATG2 morphants failed to form in 49.9% of spinal hemisegments (empty arrowhead, Fig. 3A,C) compared with MO*sp^ATG1^* morphant (18%) or COMO*sp^ATG2^* control larvae (1.1%; Fig. 3A,C). Furthermore, the dorsal nerve was thinner or absent in the majority of the hemisegments in both ATG1 (57%) and ATG2 (96%) morphants compared with controls (asterisk; COMO*sp^ATG1^*: 0%; COMO*sp^ATG2^*: 3%; Fig. 3A,D). *In vivo* time-lapse videomicroscopy showed that sMN axons of ATG1 and ATG2 morphants displayed different navigational behaviours at the horizontal myoseptum (i.e. choice point), which contrasted with the highly stereotyped behaviour of control sMN axons (Fig. 3F; Movies 5-7). As they reached this key intermediate target, a contingent of sMN axons contributing to the rostral nerve turned in the opposite direction and grew caudal to the lateral myosepta (arrowheads, Fig. 3F) in ATG1 morphants, whereas these rostrally projecting sMN axons abnormally grew ventrally beyond this choice point to ultimately fasciculate with the ventral nerve in ATG2 morphants (empty arrowheads, Fig. 3F; Movies 5-7). Notably, these axon pathfinding defects of both spastin morphants were not associated with striking muscle fibre alterations (Fig. S2). Finally, a fraction of ATG2 morphant sMN somata ectopically exited from the spinal cord and migrated along the motor tracts into the periphery, which was never observed in MO*sp^ATG1^*- or COMO*sp^ATG2^*-injected larvae (arrows, Fig. 3A,E,F; Movies 5-7). Altogether, these different sMN phenotypes of ATG1 and ATG2 morphants highlight the functional specificity of each spastin isoform in motor circuit wiring.

### DrM1 and DrM61 have crucial and non-overlapping functions in motor neuron development and zebrafish locomotion

**Fig. 4.**
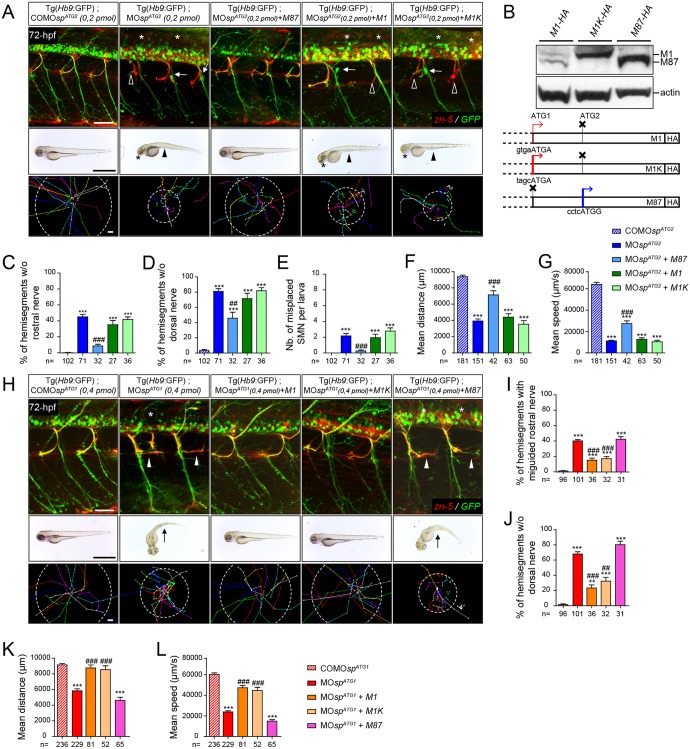
**Spastin main isoforms have non-overlapping functions in spinal motor neuron development and locomotor behaviour.** (A-G) Rescue experiments of ATG2 morphant phenotypes by M1, M1K or M87 mRNAs. (B) Western blot analysis (bottom panel) of exogenous spastin expression using an HA antibody in wild-type embryos injected with human spastin (*SPG4*) mRNAs used for rescue experiments and schematized in the upper panel. The Kozak sequence surrounding the first ATG of *M1* transcript has been replaced by an optimal consensus sequence (*M1K* mRNA) to boost M1 expression. Actin was used as a loading control. (H-L) Rescue experiments of ATG1 morphant phenotypes by *M1*, *M1K* or *M87* mRNAs. (A,H; upper panels) Immunolabelling of secondary motor neurons (sMN) in 72 hpf control, morphant and ‘rescued’ (i.e. co-injected with MO*sp^ATG1^* or MO*sp^ATG2^* together with each version of human spastin mRNAs) Tg(*Hb9*:GFP) larvae using zn-5 and GFP antibodies. (A) Arrows, empty arrowheads and asterisks indicate mispositioned sMN, missing rostral and dorsal nerves, respectively. (H) White arrowheads indicate misguided rostral nerves. Scale bars: 50 µm. (A,H; middle panels) Overall morphology of 72 hpf control, morphant and ‘rescued’ larvae. (A) Arrowheads indicate yolk tube agenesis and asterisks indicate smaller eyes. (H) Arrows indicate curved tails. Scale bars: 1 mm. (A,H; bottom panels) Tracking analysis of 72 hpf control, morphants and ‘rescued’ larvae in a touch-escape response test. Each line shows the trajectory of one larva after the stimulus. Dotted circle radius symbolizes the mean swimming covered distance. Scale bars: 1 mm. (C-G,I-L) Quantification of sMN defects (24 spinal hemisegments per larva; C-E,I,J), mean swimming distances covered (F,K) and speed (G,L) of 72-hpf control, morphant and ‘rescued’ larvae. The *n* value for each larval group is indicated under the corresponding histogram bar. *P≤0.05, **P≤0.01, ***P≤0.001 versus control larvae; ^##^*P*≤0.01, ^###^*P*≤0.001 versus morphants; Kruskal–Wallis ANOVA test with Dunn's post test. Error bars are s.e.m.

To clarify the functional specificity and redundancy of spastin main isoforms, and to confirm the relevance of both morphant phenotypes, we performed rescue and cross-rescue experiments. Human *spastin* transcripts (including their 5′UTR) mutated on the first (*M87* mRNAs) or second ATG (*M1* mRNAs) were co-injected with MO*sp^ATG1^* or MO*sp^ATG2^* morpholinos (Fig. 4). As anticipated, the overexpression of human M87 rescued the morphological, locomotion and sMN defects of ATG2 morphants, whereas human M1 failed to alleviate ATG2 morphant phenotypes (Fig. 4A,C-G). To assess whether this inability of M1 to rescue the ATG2 morphant phenotype was due to intrinsically inefficient translation from the ATG1 codon with respect to ATG2, we replaced the weak consensus Kozak sequence surrounding the ATG1 codon of human *spastin* cDNA by a robust consensus sequence (*M1K* mRNA), which increased the expression of M1 up to that of M87 (Fig. 4B). Nevertheless, higher levels of exogenous M1 still failed to rescue ATG2 morphant defects (Fig. 4A,C-G). Reciprocally, only human M1 rescued the morphological, behavioural and sMN defects of ATG1 morphants, even at low expression levels (Fig. 4H-L). These results ascertained the specificity of each *spastin* morphant phenotype and unveiled a specific role for the main spastin isoforms in vertebrate motor circuit wiring and locomotion.

### Spastin CRISPR/Cas9 mutants develop similar locomotor and SMN defects as ATG1 and ATG2 morphant larvae

**Fig. 5.**
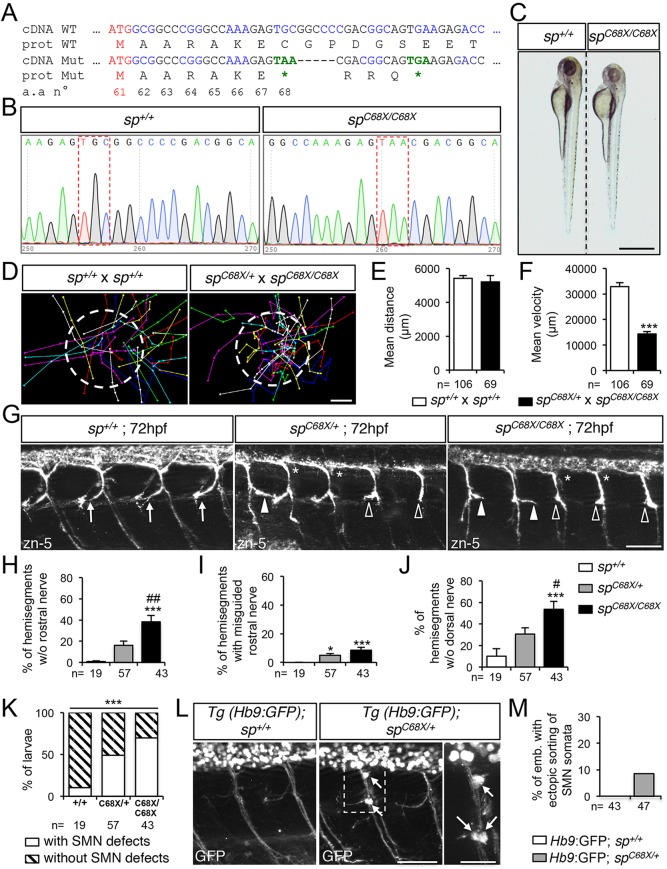
**Spastin CRISPR/Cas9 mutants show overlapping locomotor and SMN phenotypes with ATG1 and ATG2 morphants.** (A) mRNA and protein sequences of wild-type (WT) and *sp^C68X^* CRISPR/Cas9 mutant (Mut). The second ATG codon (Met 61) and premature stop codons (*) are indicated in red and green, respectively. Black and blue letters are used to discriminate two successive codons. Numbers indicate amino acid position. (B) Sequence analysis of wild-type (*sp^+/+^*) and *sp^C68X/C68X^* mutant DNA. Red dashed boxes show the nucleotide substitution generating a premature STOP codon. (C) Overall morphology of 72 hpf *sp^+/+^* and *sp^C68X/C68X^* larvae*.* Scale bar: 6 mm. Some mutant larvae are smaller than their control siblings. (D-F) Touch-evoked escape behaviour of 72 hpf larvae obtained from crosses between *sp^C68X/C68X^* and *sp^C68X/+^* or control *sp^+/+^*. (D) Each line represents the trajectory of one larva after touch stimulation. Dotted-circle radius symbolises the mean swimming distance covered by all larvae. The distance between two dots represents the distance covered by a larva between two consecutive frames. Scale bar: 4 mm. (E,F) Quantification of the mean swimming distance (E) and mean swimming speed (F). (G) Immunolabelling of sMN in *sp^+/+^*, *sp^C68X/+^* and *sp^C68X/C68X^* larvae using zn-5 antibody. Arrows indicate control rostral nerves, while white arrowheads, empty arrowheads and asterisks indicate misguided rostral nerve, missing rostral nerves and dorsal nerves of *sp^C68X^* mutant larvae, respectively. (H-J) Quantification of sMN defects in 72 hpf *sp^+/+^*, *sp^C68X/+^* and *sp^C68X/C68X^* larvae. Quantifications were carried out on 24 spinal hemisegments per larva. (K) Penetrance of SMN defects. (L) Immunolabelling of SMN in 72 hpf *sp^+/+^* or *sp^C68X/+^* Tg(*Hb9*:GFP) larvae using a GFP antibody. Arrows indicate mispositioned SMN somata. (M) Percentage of larvae with ectopic sorting of SMN somata outside the spinal cord. (E,F,H-K,M) The number (*n*) of larvae analysed per condition is indicated under the corresponding histogram bar. *P≤0.05, ***P≤0.001 versus internal controls; ^#^*P*≤0.05, ^##^*P*≤0.01 versus *sp^C68X/+^* larvae; non-parametric Mann–Whitney test (E,F); Kruskal–Wallis ANOVA test with Dunn's post test (H-J) and χ^2^ test (K). Error bars are s.e.m. (G,L) Lateral views of the trunk, anterior towards the left. Scale bars: 50 µm.

To further strengthen the specificity of the phenotypes associated with the use of MO*sp^ATG1^* or MO*sp^ATG2^* morpholinos, we generated a spastin CRISPR/Cas9 genetic mutant harbouring a truncating mutation after the second ATG codon (C68X; Fig. 5A-B), thereby impeding the synthesis of both M1 and M61 spastin isoforms. The vast majority of *sp^C68X/C68X^* homozygous mutants appeared smaller than their control siblings (Fig. 5C) and showed reduced swimming speed in a touch-escape response test (Fig. 5D-F; Movies 8,9) as ATG1 and ATG2 morphant larvae (Fig. 2F). Furthermore, both *sp^C68X/+^* and *sp^C68X/C68X^* mutants exhibited pathfinding defects of sMN axons, consisting of a combination of the axon-targeting errors described in ATG1 (i.e. abnormal caudal turning of the rostral nerve; white arrowheads, Fig. 5G) and ATG2 (missing rostral and dorsal nerves; empty arrowheads and asterisks, respectively, Fig. 5G) morphants, and which were more severe in homozygous than in heterozygous mutants (Fig. 5H-K). However, the penetrance of these axon pathfinding defects was incomplete in both *sp^C68X/+^* and *sp^C68X/C68X^* mutants (Fig. 5K). Furthermore, crossing *sp^C68X/C68X^* mutants with Tg(*Hb9*:GFP) fish led to misrouted SMN somata outside the spinal cord in 8.2% of the progeny (a phenotype also observed in ATG2 morphants) compared with 0% in the crosses between wild-type and Tg(*Hb9*:GFP) fish (arrows, Fig. 5L,M). The strong similarities between ATG1, ATG2 morphant and *sp^C68X^* mutant phenotypes, as well as the dose-sensitivity of SMN development to spastin expression levels largely exclude the possibility that the observed defects could be caused by non-specific effects of morpholino injection or Cas9 genome engineering rather than by the loss of spastin function, thereby validating both loss-of-function approaches.

### DrM1 controls sMN axon targeting by inhibiting the BMP pathway

**Fig. 6.**
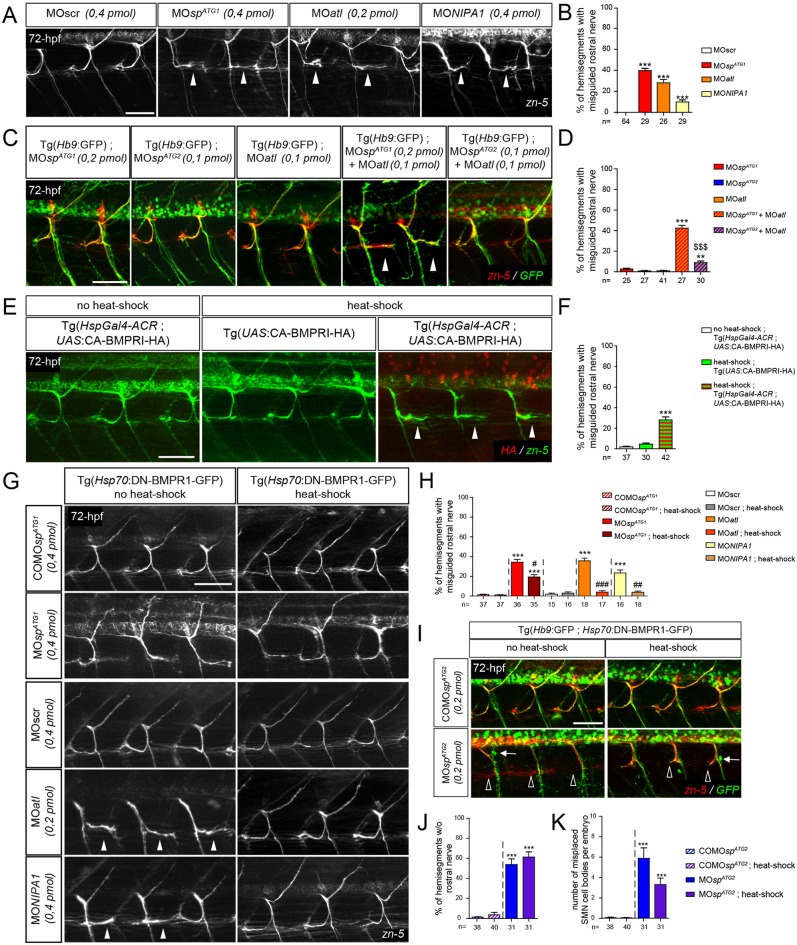
**DrM1 controls secondary motor axon targeting through BMP inhibition as atlastin 1 and NIPA1 do.** (A,C,E,G,I) Immunolabelling of secondary motor neurons (sMN) at 72 hpf using zn-5 (A,G), zn-5 and HA (E), or zn-5 and GFP antibodies (C,I). Lateral views of the trunk, anterior towards the left. White arrowheads, empty arrowheads and arrows indicate misguided rostral nerves, missing rostral nerves and misplaced SMN somata, respectively. (B,D,F,H) Mean percentage of misguided rostral nerves. (J) Mean percentage of hemisegments without rostral nerves. (K) Mean number of misplaced SMN somata per embryo. (B,D,F,H,J,K) Quantifications were performed on 24 spinal hemisegments per larva. The *n* value for each larval group is indicated under the corresponding histogram bar. **P≤0.01, ***P≤0.001 versus internal controls; ^$$$^*P*≤0.001 for co-injection comparison; ^#^*P*≤0.05, ^##^*P*≤0.01, ^###^*P*≤0.001 versus morphant larvae; Kruskal–Wallis ANOVA test with Dunn's post test. Error bars are s.e.m. (A,B) sMN analysis in larvae injected with MO*sp^ATG1^*, MO*atl*, MO*nipa1* or control (MOscr) morpholinos. (C,D) sMN analysis in larvae injected with sub-efficient doses of MO*sp^ATG1^*, MO*sp^ATG2^* or MO*atl* morpholinos or co-injected with the same sub-efficient doses of MO*atl* and MO*sp^ATG1^* or MO*sp^ATG2^* morpholinos. (E,F) sMN analysis in non-heat-shocked or heat-shocked Tg(*HspGal4-ACR*;*UAS*:CA-BMPRI-HA) and heat-shocked Tg(*HspGal4-ACR*) transgenic larvae. (G-K) sMN analysis in non-heat-shocked or heat-shocked Tg(*Hsp70*:DN-BMPR1-GFP) transgenic larvae injected with MO*sp^ATG1^*, MO*atl*, MO*nipa1*, MO*sp^ATG2^* and appropriate control morpholinos. Scale bars: 50 µm.

M1 spastin was shown to interact with the HSP-causing protein atlastin 1 (Evans et al., 2006; Sanderson et al., 2006), which controls zebrafish SMN axon pathfinding through the inhibition of the BMP pathway (Fassier et al., 2010). Furthermore, atlastin 1 binds to the HSP protein NIPA1 (Rainier et al., 2003; Botzolakis et al., 2011), which was equally identified as a BMP inhibitor (Wang et al., 2007; Tsang et al., 2009). To investigate whether defective BMP signalling could underlie the characteristic pathfinding defect of the rostral nerve in ATG1 morphants, we first analysed sMN development in 72 hpf larvae injected with morpholinos targeting the translation initiation site of either the *atlastin 1* (*atl1*) (Fassier et al., 2010) or *nipa1* transcripts, or with a universal control morpholino (MOscr). Interestingly, the knockdown of each of these BMP inhibitors similarly altered the pathfinding of the rostral nerve, which abnormally grew caudally along the horizontal myoseptum as described for ATG1 *spastin* morphants (arrowheads, Fig. 6A,B). Furthermore, the co-injection of MO*atl* and MO*sp^ATG1^* at subefficient doses, which did not affect sMN development when injected separately, increased massively the number of misguided rostral nerves (40%) compared with the co-injection of MO*atl* and MO*sp^ATG2^* (5%) (arrowheads, Fig. 6C,D), suggesting that the sMN defects caused by the lack of DrM1 spastin and atlastin 1 may involve interference with a common mechanistic pathway. To determine whether an upregulation of the BMP pathway, independently of any variation in HSP protein expression, was sufficient to induce the aberrant caudal turning of the rostral nerve, we generated a novel Tg(*UAS*:CA-BMPRI-HA) transgenic line expressing a constitutively active version of type Ia BMP receptor under the control of a *UAS* promoter, based on a published line (Quillien et al., 2011). To temporally control the upregulation of BMP signalling, we crossed these fish with the Tg(*HspGal4-ACR*) line expressing the transactivator GAL4 under the control of a heat-shock inducible promoter. We then heat-shocked the progeny at 42 hpf, when sMN axons composing the rostral nerve reached the horizontal myoseptum and were subsequently guided towards their appropriate targets. Heat-shocked Tg(*HspGal4-ACR*; *UAS*:CA-BMPRI-HA) transgenic larvae showed caudally oriented misguided rostral nerves (26%) compared with both heat-shocked Tg(*UAS*:CA-BMPRI-HA) and non-heat-shocked Tg(*HspGal4-ACR*; *UAS*:CA-BMPRI-HA) control larvae (arrowheads, Fig. 6E,F). This test revealed that the over-activation of BMP signalling altered sMN axon targeting in a similar way to the loss of DrM1 spastin, atlastin 1 or NIPA1. We finally assessed whether a genetic inhibition of the BMP pathway could alleviate the sMN defects of *spastin* ATG1 or *nipa1* morphants as previously shown for *atl1* knockdown (Fassier et al., 2010). To this aim, MO*sp^ATG1^*, COMO*sp^ATG1^*, MO*nipa1* and MO*atl* morpholinos were injected in Tg(*Hsp70*:DN-BMPR1-GFP) transgenic embryos expressing a dominant-negative version of type-Ia BMP receptor under the control of a heatshock-inducible promoter (Pyati et al., 2005). ATG1, *nipa1* and *atl1* morphant Tg(*Hsp70*:DN-BMPR1-GFP) larvae, which were subjected to heat shock at 42 hpf, showed a striking reduction in the number of mistargeted rostral nerves at 72 hpf compared with non-heat-shocked morphant siblings or heat-shocked control larvae (44%, 83.7% and 89% reduction, respectively; Fig. 6G-H). In contrast, the inhibition of BMP signalling had no beneficial impact on ATG2 morphant sMN phenotypes (Fig. 6I-K). Altogether, these results showed a specific role for DrM1 spastin in sMN axon targeting via the inhibition of the BMP pathway and provide *in vivo* evidence for a similar role of the HSP gene *nipa1* in vertebrate motor neuron development.

### DrM61 acts downstream of neuropilin 1a signalling in spinal motor neurons

**Fig. 7.**
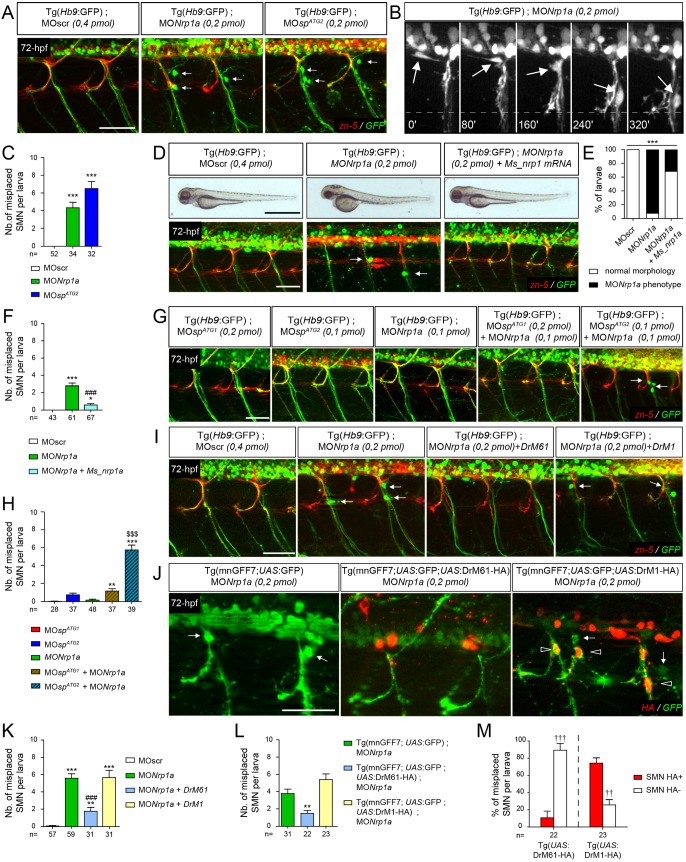
**DrM61 acts as a downstream effector of neuropilin 1a signalling in motor neurons.** (A-C) Neuropilin 1a (*nrp1a*) morphants show misrouted motor neuron somata similar to ATG2 morphants. (A) Analysis of secondary motor neurons (sMN) in 72 hpf Tg(*Hb9*:GFP) transgenic larvae injected with MO*nrp1a*, MO*sp^ATG2^* or MOscr morpholinos using zn-5 and GFP antibodies. (B) Representative stills of time-lapse recordings showing SMN behaviour in 40-72 hpf Tg(*Hb9*:GFP) transgenic larvae injected with *Nrp1a* (*n*=5 larvae; *n*=32 spinal hemisegment monitored). Arrows track the aberrant migration of SMN somata along motor axon tracts of *nrp1a* morphants. Scale bar: 10 µm. (D-F) Rescue experiments of *nrp1a* morphant phenotypes with mouse *Nrp1* mRNA (Ms_nrp1 mRNA). (D) Overall morphology (upper panels) and SMN analysis (bottom panels) of 72 hpf larvae injected with MOscr, MO*Nrp1a* or MO*Nrp1a* and Ms_*nrp1* mRNA. (E) Distribution of wild-type and *nrp1a* morphant phenotypes in each injection group. ***P≤0.001; χ^2^ test. (F) Mean number of misplaced SMN somata per larvae. (G) SMN analysis in 72 hpf Tg(*Hb9*:GFP) transgenic larvae injected with sub-efficient doses of MO*sp^ATG1^*, MO*sp^ATG2^* or MO*Nrp1a* or co-injected with the same sub-efficient doses of MO*Nrp1a* and MO*sp^ATG1^* or MO*sp^ATG2^* morpholinos. (I) SMN analysis in 72 hpf Tg(*Hb9*:GFP) transgenic larvae injected with control (MOscr) or MO*Nrp1a* morpholinos or co-injected with MO*Nrp1a* and *DrM1-* or *DrM61-spastin* mRNA. (J) SMN analysis in 72 hpf Tg(mnGFF7;*UAS*:GFP), Tg(mnGFF7;*UAS*:GFP;*UAS*:DrM61-HA) and Tg(mnGFF7; *UAS*:GFP;*UAS*:DrM1-HA) transgenic larvae using HA and GFP antibodies. (A,D,G,I,J) Lateral views of the trunk, anterior towards the left. Arrows indicate mispositioned SMN somata. Scale bars: 50 µm. (J) Empty arrowheads indicate misplaced HA-positive SMN. (C,F,H,K,L) Mean number of mispositioned SMN per larva (quantified on 24 spinal hemisegments/larva). (M) Percentage of misplaced HA-positive or -negative SMN per larva (quantified on 24 spinal hemisegments/larva). (C,F,H,K-M) The number of larvae or SMN analysed per group (*n*) is mentioned under each corresponding histogram bar. *P≤0.05, **P≤0.01, ***P≤0.001 versus internal controls; ^$$$^*P*≤0.001 versus single injection of each morpholino; ^###^*P*≤0.001 versus morphant larvae; ^††^*P*≤0.01, ^†††^*P*≤0.001 HA-positive versus -negative SMN; Kruskal–Wallis ANOVA test with Dunn's post test. Error bars are s.e.m.

To unravel the signalling pathway(s) in which spastin short isoform is involved during SMN development, we focused on the migratory motor neuron phenotype (i.e. the aberrant exit of SMN cell somata from the spinal cord), which was exclusively observed in ATG2 morphants. We searched for similar migratory defects in published mutants or morphants for signalling cues and associated receptors known to be involved in neuronal migration and axon guidance processes. Notably, the knockdown of the semaphorin receptor neuropilin 1a (Nrp1a) was shown to cause the misrouting of SMN cell somata from the spinal cord into the periphery (Feldner et al., 2005). Immunolabelling and time-lapse videomicroscopy of Tg(*Hb9*:GFP) transgenic larvae injected with *nrp1a* morpholino (MO*nrp1a*; Feldner et al., 2005) confirmed that Nrp1a-depleted SMN somata abnormally migrated into the periphery along spinal motor tracts (arrows, Fig. 7A-C; Movie 10), as shown for ATG2 morphant SMNs (Fig. 3F; Movie 7). Co-injection of mouse *Nrp1* mRNA together with a zebrafish *nrp1a* morpholino rescues both the morphological and SMN migratory defects of *nrp1a* morphants, reinforcing the specificity of these phenotypes (Fig. 7D-F). Moreover, the double knockdown of Nrp1a and DrM61 spastin isoform using sub-efficient doses of MO*nrp1a* and MO*sp^ATG2^* morpholinos dramatically increased the number of mispositioned SMN somata, compared with the co-injection of MO*nrp1a* and MO*sp^ATG1^* or to the single injection of each morpholino (Fig. 7G-H). To determine whether DrM61 spastin could act downstream of the Nrp1a receptor, we carried out rescue experiments by overexpressing DrM61 spastin, either ubiquitously or selectively in SMNs, in an *nrp1a* morphant context. To achieve this, *nrp1a* morpholino (MO*nrp1a*) was co-injected with *DrM61spastin-HA* mRNA (Fig. 7I,K) in Tg(*Hb9*:GFP) embryos or injected in triple transgenic Tg(mnGFF7; *UAS*:GFP; *UAS*:DrM61-HA) embryos specifically expressing DrM61 spastin and GFP in SMNs (Fig. 7J,L-M). Importantly, both the ubiquitous and SMN-targeted overexpression of DrM61 spastin partially rescued the aberrant exit of *nrp1a* morphant SMN somata from the spinal cord. In contrast, the overexpression of DrM1 in the *nrp1a* morphant background failed to alleviate these migratory defects (Fig. 7I,K). Furthermore, although 74% of ectopic SMN somata in MO*nrp1a*-injected Tg(mnGFF7; *UAS*:GFP; *UAS*:DrM1-HA) larvae were HA positive, this rate dropped to 11% in MO*nrp1a*-injected Tg(mnGFF7; *UAS*:GFP; *UAS*:DrM61-HA) larvae (empty arrowheads; Fig. 7J,L-M). This suggested that most neurons that failed to be rescued by DrM61-HA did not express the rescuing transgene, which might be due to the intrinsic variegation in transgene expression, frequently occurring in transgenic zebrafish. These data thus unveiled a specific role for DrM61 as a downstream effector of Nrp1a signalling in spinal motor neurons.

## DISCUSSION

This *in vivo* functional analysis of the main spastin isoforms reveals their crucial and specific involvement in two distinct developmental signalling pathways that are both essential for motor circuit wiring and locomotion in vertebrates. Here, we provide evidence for a concerted role for DrM1 spastin and the HSP-causing proteins atlastin 1 and NIPA1 in the inhibition of the BMP pathway and unveil a specific role for DrM61 spastin as a downstream effector of neuropilin 1a signalling in developing motor neurons.

### Multiplying spastin function through alternative translation

We and others have previously shown that the dysregulation of microtubule (MT) dynamics associated with the lack of spastin MT-severing activity is crucial for motor axon development and/or homeostasis in invertebrate and vertebrate model organisms (Sherwood et al., 2004; Trotta et al., 2004; Tarrade et al., 2006; Wood et al., 2006; Fassier et al., 2013; Denton et al., 2014; Havlicek et al., 2014; Brill et al., 2016). However, owing to the growing interest in this major HSP protein, the characterisation of its function in various cellular processes has progressively added to the richness of its functional diversity but simultaneously complicated the comprehensive understanding of *SPG4*-linked HSP physiopathology. Indeed, the main spastin isoforms have so far been involved in many cellular processes, including ER shaping (M1; Park et al., 2010), lipid droplet homeostasis (M1; Papadopoulos et al., 2015) and endosomal tubule fission (M1 and M87; Allison et al., 2013, 2017), although the causal connection between their dysfunction and HSP axonopathy remains to be determined. A major challenge in the field therefore aims to decipher the individual function(s) of each spastin isoform and to identify the HSP-causing culprit(s) among them. To tackle this key issue, we undertook the first comparative loss-of-function analysis of spastin main isoforms *in vivo*, using a morpholino-based strategy to block their respective synthesis from each initiation codon during zebrafish development. Our study provides a proof of concept that morpholino-based knockdown analyses coupled with appropriate rescue experiments and relevant controls (Stainier et al., 2017), including a newly generated *sp^C68X^* mutant, represent an appropriate approach to block the expression of protein isoforms resulting from alternative translation, unlike conventional si- or sh-RNA gene silencing technologies that mostly trigger the degradation of the targeted transcript (Summerton, 1999). This strategy allowed us to unmask a crucial and specific role for each spastin isoform in motor circuit wiring and larval locomotion. Although M1 has previously been shown to carry out selective cellular functions through its exclusive transmembrane hairpin domain (Park et al., 2010; Papadopoulos et al., 2015), we here identify a specific role for the N-terminally truncated isoform DrM61 in zebrafish SMN migration and axon targeting, which is supported by: (1) its contrasted subcellular distribution with respect to DrM1; (2) the marked differences in SMN and behavioural defects between ATG1 and ATG2 morphants; and (3) the inability of M1 to rescue the phenotypes of ATG2 morphants. Furthermore, M1 has so far been suggested to play a major role in HSP pathogenesis (Solowska et al., 2008, 2014; Leo et al., 2017). However, our loss-of-function analysis, mimicking the haploinsufficiency associated with the vast majority of *SPG4* mutations (Fonknechten et al., 2000; Roll-Mecak and Vale, 2008; Riano et al., 2009), establishes that the depletion of DrM61, independent of any impact on DrM1, is at least as detrimental for developing motor neurons as the depletion of DrM1. Altogether, our data thus demonstrate that alternative translation of the *SPG4* transcript and the partly resulting multifunctionality of spastin are required for vertebrate motor circuit wiring. These results also suggest that the haploinsufficiency of each spastin isoform may concomitantly alter motor circuit formation and connectivity through distinct pathogenic pathways.

### DrM61 acts downstream of neuropilin 1a signalling

Our analysis of DrM61 during zebrafish development identifies a specific role for this short isoform as a downstream effector of the neuropilin 1a (Nrp1a) receptor in motor neurons. DrM61 is required for Nrp1a-mediated inhibition of SMN somata migration outside the zebrafish spinal cord, a cellular process involving both semaphorin 3A and VEGF ligands (Feldner et al., 2005). Importantly, the signalling pathways induced by the interactions between these cues and the Nrp1 receptor have been involved in mammalian motor circuit development and maintenance, such as motor neuron somata migration (Schwarz et al., 2004; Lee et al., 2015), axon pathfinding and fasciculation (Jouet et al., 1994; Castellani et al., 2000; Huber et al., 2005; Huettl et al., 2011), and motor neuron survival (Oosthuyse et al., 2001). Furthermore, they recently emerged as key pathogenic pathways in two other neurodegenerative disorders affecting upper and/or lower motor neurons (Venkova et al., 2014; He et al., 2015). Altogether, these data may therefore imply that defective Nrp1 signalling associated with M87 deficiency may contribute to *SPG4*-linked HSP by altering upper motor tract development and/or homeostasis. How would M87 regulate Nrp1-mediated pathways? As M87 exhibits an increased severing activity compared with M1 (Solowska et al., 2008), one possible explanation could be that M87 participates in the remodelling of the microtubule cytoskeleton required for Nrp1-mediated motor neuron migration and/or axon targeting. However, as M87 spastin was shown to be sufficient to rescue the endosomal tubulation phenotype of cells lacking spastin (Allison et al., 2013), a role for this short isoform in Nrp1 endosomal trafficking could also been considered. Further investigations will thus be required to clarify the molecular mechanisms underlying M87 functional specificity.

### DrM1 is a BMP inhibitor

Over the past decades, several studies in invertebrate models revealed the key regulatory role of the BMPs in motor axon guidance (Colavita et al., 1998) and synapse formation at the neuromuscular junction (Bayat et al., 2011). Furthermore, the over-activation of BMP signalling has progressively emerged as a common pathogenic mechanism in five genetic subtypes of HSP (Wang et al., 2007; Fassier et al., 2010; Renvoisé et al., 2012; Nahm et al., 2013; Song et al., 2013; Mao et al., 2015). This dual involvement of BMP signalling in both motor neuron development and degeneration raised new issues on the timing of the initial pathogenic defects causing HSP. Notably, defective BMP signalling was suggested to contribute to *SPG4*-linked HSP based on the increased phosphorylation of its canonical readout Smad1/5, which was associated with spastin knockdown in HeLa cells (Tsang et al., 2009). We here confirmed these *in vitro* findings by unravelling a specific role for M1 as a BMP inhibitor required for vertebrate motor circuit wiring. DrM1 morphants or *sp^C68X^* mutants exhibit SMN axon pathfinding defects that strikingly mimic those associated with: (1) the depletion of HSP-causing BMP inhibitors atlastin 1 and NIPA1; or (2) the overexpression of a constitutively active version of BMPRI; and (3) are partially rescued by the genetic inhibition of BMP signalling. Moreover, our work revealed a cooperative role for two key AD-HSP partners, atlastin 1 and spastin, in the inhibition of the BMP pathway, which further supports the key role of BMP signalling in HSP physiopathology. How would M1 spastin participate in the regulation of this pathway? NIPA1 and atlastin 1 have been shown to modulate BMP signalling through the regulation of BMP receptor trafficking (Wang et al., 2007; Tsang et al., 2009; Nahm et al., 2013; Zhao and Hedera, 2013). Furthermore, both spastin-depleted HeLa cells and motor neuron growth cones of ATG1 morphants exhibit abnormal endosomal tubulation, which was associated with defective trafficking of transferrin receptors in the mammalian cells (Allison et al., 2013). Therefore, M1 most likely acts in concert with these HSP proteins to control BMP receptor trafficking and plasma membrane concentration, which may in turn influence motor neuron responses to extracellular BMP signals. Interestingly, BMP signalling has been shown to regulate MT stability and axonal transport homeostasis in *Drosophila* motor neurons (Wang et al., 2007; Nahm et al., 2013), two crucial processes in spastin-related HSP pathogenesis (Tarrade et al., 2006; Fassier et al., 2013; Denton et al., 2014; Havlicek et al., 2014).

In conclusion, the functional analyses of genes responsible for neurodegenerative disorders have gradually unveiled crucial molecular links between neurodegenerative processes and key developmental signalling pathways mediated by axon guidance molecules (Bai et al., 2011; Van Hoecke et al., 2012; Venkova et al., 2014; He et al., 2015). Although their role as neuronal circuit wirers has been well established, the dissection of their contribution to neurodegenerative diseases represents an important area for future research in the field, which would provide priceless information on their potential use as tractable therapeutic targets.

## MATERIALS AND METHODS

### Zebrafish care and maintenance

Zebrafish embryos (*Danio rerio*) were obtained from natural spawning of wild-type or transgenic Tg(*Hb9*:GFP) (Flanagan-Steet et al., 2005), Tg(mnGFF7;*UAS*:GFP) (Asakawa et al., 2008, 2013), Tg(*Hsp70*:DN-BMPR1-GFP) (Pyati et al., 2005), Tg (*HuC*:Gal4; *UAS*:myr-Venus) (Baraban et al., 2013), the newly generated Tg(*UAS*:DrM1-Spastin-HA), Tg(*UAS*:DrM61-Spastin-HA), Tg(*UAS*:CA-BMPRI-HA) derived from the published line (Quillien et al., 2011), Tg(*HspGal4-ACR)*, and the spastin CRISPR/Cas9 *sp^C68X^* mutant lines. All embryos were maintained at 28°C in E3 medium and staged by hour post-fertilisation (hpf) and gross morphology according to Kimmel et al. (1995). Pigment formation was prevented by adding 0.2 mM of 1-phenyl-2-thiourea (PTU, Sigma) to the E3 media after the prim-5 stage. To induce transgene expression from *hsp* promoters, larvae were heat-shocked for 60 min at 40°C in pre-warmed E3 medium using a thermomixer (Eppendorf).

All our experiments were performed in agreement with the European Directive 210/63/EU on the protection of animals used for scientific purposes, and the French application decree ‘Décret 2013-118’. The fish facility has been approved by the French ‘Service for animal protection and health’, with the approval number A-75-05-25.

### Constructs and generation of transgenic and mutant lines

Zebrafish *spastin* cDNA was obtained from the IMAGE clone 4725923, including 121 bases upstream of the ATG initiation codon. It was subsequently amplified and HA-tagged by PCR and cloned into pCS2+ using the *Xho*I/*Xba*I restriction sites (pCS2+DrSp-HA). Targeted mutagenesis of the first ATG was performed using the megaprimer PCR method (pCS2+DrSp-HAMut^ATG1^) while the mutagenesis of the second ATG codon was carried out using the QuickChange site-directed mutagenesis kit (Stratagene) and a set of complementary primers (pCS2+DrSp-HAMut^ATG2^).

The DNA constructs used to generate the Tg(*UAS*:DrM1-HA) and Tg(*UAS*:DrM61-HA) transgenic lines were composed by assembling the three elements below in the following order: (1) a *Sac*II-*Swa*I fragment of plasmid ‘T2 US E1B Cit UAS E1B MCS ACR’ (a kind gift from Sebastian Gerety and David Wilkinson, Neural Development Laboratory, The Francis Crick Institute, London, UK) composed of an ‘alpha-crystallin:mRFP’ cassette for screening purposes; (2) a *Swa*I-*Avr*II fragment containing a promoter module made of five UAS sites followed by a minimal promoter and a short leader sequence (Baraban et al., 2013); and (3) a *Spe*I-*Not*I fragment containing the full-length DrSp-HAMut^ATG1^ or DrSp-HAMut^ATG2^ cDNAs followed by the SV40 3′UTR and polyadenylation signal of the pCS2+ vector. These resulting constructs were cloned into pBluescrit SKI-*Sce*I (Grabher et al., 2004) to yield (*UAS*:DrM61-HA) and (*UAS*:DrM1-HA) plasmids, and were injected at 30 pg/egg into freshly fertilised zebrafish eggs together with the I-*Sce*I endonuclease (Roche). Injected fish were raised to adulthood and screened for germline transmission by detecting the red fluorescence in the lens of their progeny at 72 hpf. The Tg(*UAS*:DrM1-HA) and Tg(*UAS*:DrM61-HA) transgenic lines were established from these positive carriers.

For the Tg(*UAS*:CA-BMPRI-HA) line, the constitutively active form of BMP type-1a receptor (Nikaido et al., 1999) was PCR-amplified and HA-tagged from a ‘pME-MCS-ca-BMPR1-HA’ construct (a kind gift from Elise Cau and Patrick Blader, Centre de Biologie Intégrative, Université Toulouse III - Paul Sabatier, Toulouse, France; Quillien et al., 2011) using the following primers: *Avr*II-zBMPRIa-CA_FOR: 5′-TACGCGCCTAGGCAATTTGACAATGCGTCAGC-3′ and *Not*I-HA-zBMPRIa-CA_REV: 5′-TTTTCCTTTTGCGGCCGCTCAAGCGTAATCTGGAACATCGTATGGTGAGATTTTAATGTCTTGAGATTCC-3′, and cloned in place of *spastin* cDNA in the (*UAS*:DrM61-HA) plasmid using the *Avr*II/*Not*I restriction sites. Transgenic fish were generated and identified as described above. The Tg(*HspGal4-ACR*) line was generated in David Wilkinson's lab.

Human spastin cDNA mutated on the second ATG (encoding M1) was obtained from a pIRESspastin-m87 construct provided by E.R.'s lab (Allison et al., 2013). As this construct had no 5′UTR, which is required for the regulation of spastin isoform ratio, we amplified a 245 bp fragment corresponding to human *SPG4* 5′UTR from human genomic DNA [kindly provided by Catalina Betancur, Sorbonne Universités, Neuroscience Paris Seine - Institut de Biologie Paris-Seine (NPS-IBPS), Paris, France]. and cloned it in pIRESspastin-m87 using the *Eco*RI site located upstream *SPG4* first initiation codon (primers Nhe-*Avr*II-spastinUTR_FOR: 5′-ATTGCTAGCCTAGGCCCGAGCCACCGACTGCAGG-3′ and Eco-spastinUTR_REV: 5′-GGCGAATTCATTCACAGCTCTCACTGCC-3′). M1-spastin cDNA including the 5′UTR was next subcloned in place of the zebrafish spastin cDNA in the (*UAS*:DrM61-HA) plasmid using *Avr*II/*Not*I restriction sites to yield the (*UAS*:M1-HA) construct. In the (*UAS*:M1K-HA) construct, the 5′UTR sequence was removed and the initiation codon was placed immediately after the 5′-ACTTTGAGCTCCTCCACACGAATTGCTAGC-3′ Kozak leading sequence, which provides a more favourable translation initiation context. (*UAS*:M87-HA) was generated by targeted mutagenesis of the (*UAS*:M1-HA) construct using the megaprimer PCR method. All human spastin constructs were subcloned in the pCS2+ vector and *in vitro* transcribed using the SP6 mMessage mMachine kit (Ambion).

The *sp^C68X^* mutant was generated by TEFOR-AMAGEN (UMS CNRS 3504/INRA 1374). Cas9 protein and sgRNA targeting a region downstream of the second ATG codon (sgRNA364: 5′-GCCAAAGAGTGCGGCCCCGA-3′) were synthesised by Tacgene facility. Wild-type TU embryos (*n*=45) were injected at the one-cell stage with 1 nl of a solution containing 19 µM of Cas9 protein and 50 ng/µl of sgRNA364. To test sgRNA efficiency, genomic DNA was isolated from seven embryos and PCR amplified with the Dream Taq DNA Polymerase (ThermoFisher Scientific) using primers flanking the Cas9-targeted region (FOR: 5′-CAAACCAGAGAGCCGGACA-3′; REV: 5′-GCTTGTTTGTGGTAGTTTCGGA-3′). PCR products were analysed by capillary electrophoresis with the LabChip 1K DNA assay (Perkin Elmer). Under these conditions, 100% of the embryos injected with the sgRNA364 were mutants. Founders were then outcrossed with the wild-type TU strain and the resulting F1 embryos were screened for frameshift mutations by PCR (as mentioned above) and Sanger sequencing. A founder fish harbouring a premature stop codon at amino acid 68 was selected and outcrossed to wild-type mates to create the *sp^C68X^* mutant line. Heterozygous carriers of the mutant allele (*sp^C68X/+^*) were then incrossed to generate homozygous mutant larvae (*sp^C68X/C68X^*).

### Morpholinos and RNA injections

Morpholino oligonucleotides (MO) targeting each translation start site of zebrafish *spastin* mRNA and *nipa1* mRNA, as well as control mismatch or universal MOs, were developed by GeneTools and designed as follows: MO*sp^ATG1^*, 5′-ATTCATTCACCCTTCTCGGGCTCTC-3′; MO*sp^ATG^*, 5′-GCTGAAACAGCCACCGAAGAAGCC-3′; COMO*sp^ATG1^*, 5′-ATTGATTCAGCCTTGTCGCGCTGTC-3′; COMO*sp^ATG2^*, 5′-GCTCAAACACCCAGCGAACAAGGC-3′; MO*nipa1*: 5′-GGGTCTCGTCCATAAATATGTGCGA-3′; and MOscr, 5′-CCTCTTACCTCAGTTACAATTTATA-3′.

MOs were injected at two-cell stage. MO*sp^ATG1^*, COMO*sp^ATG1^* and MO*nipa1* were injected at 0.4 pmol/embryo while MO*sp^ATG2^*, COMO*sp^ATG2^*, MO*atl* (Fassier et al., 2010), MO*nrp1a* (Feldner et al., 2005) and MOscr were injected at 0.2 pmol/embryo. For rescue experiments, mouse *Nrp1* cDNA (Nrp1-201: ENSMUST00000026917.9) was reverse transcribed, PCR amplified (forward primer, 5′-ATCCGGAATTCATGGAGAGGGGGCTGCCGTTGC-3′; reverse primer, 5′-CTAGTCTAGATTAAGCGTAATCTGGAACATCGTATGGGTACGCCTCTGAGTAATTACTCTGTGGG-3′) and HA-tagged using the SuperScript III One-Step RT-PCR System with Platinum Taq High Fidelity (ThermoFisher Scientific) from a total RNA extract of E16.5 mouse embryonic brain following the manufacturer's instructions. The PCR product was subsequently cloned in the pCS2+ vector using *Eco*RI and *Xba*I restriction sites. Human spastin and mouse *Nrp1* mRNAs were *in vitro* transcribed from linearised pCS2+ constructs using the SP6 mMessage mMachine kit (Ambion) and injected at the one-cell stage at 200 pg per embryo.

### Touch-escape response test and manual tracking

Locomotor behaviour of control, *spastin* mutant, morphant and rescued larvae was assessed at 72 hpf by performing a touch-escape response test. A tactile stimulus was applied on each larva with a pair of forceps and their escape behaviour was recorded under a Leica M165 C binocular stereomicroscope equipped with a Leica IC80 HD camera. Swimming speed and covered distance of each larva were quantified using the Manual Tracking plug-in (ImageJ software). Stereotypic movement patterns of the touch-evoked startle response were recorded under a Leica M165 FC binocular stereomicroscope equipped with a DFC 345 FX camera. C-bend maximal body curvature angles were manually scored from the videos as described previously (Jain et al., 2014).

### Whole-mount immunohistochemistry

Zebrafish embryos or larvae were fixed in 4% paraformaldehyde for 2 h at room temperature, washed with PB-T1% (1% Triton X-100 in PBS), permeabilised in a 0.25% trypsin solution (at 25°C) after 24 hpf, blocked for 2 h in PB-T1% supplemented with 10% of normal goat serum and subsequently incubated overnight with the following antibodies: zn-5 (1:200, ZIRC, University of Oregon), GFP (1:1000, A11122, Molecular Probes), HA (1:100, 11867423001, Roche), F59 and F310 (1:100, Developmental Studies Hybridoma Bank, F59 and F310 was deposited to the DSHB by F. E. Stockdale). After several washes in 1% PBT, larvae were incubated overnight at 4°C with Alexa Fluor 488- or 555-conjugated goat anti-mouse, -rabbit or -rat antibodies (1:1000, Molecular Probes). For sMN and muscle fibre analysis, images were acquired using a fluorescence microscope equipped with an Apotome module (Zeiss, Axiovert 200M) and a 20× air objective (NA 0.5) and processed with the ImageJ software. Images of spastin isoform distribution in SMN were acquired using a confocal laser-scanning microscope (TCS SP5 Acousto-Optical Beam Splitter; Leica) and a 63× oil immersion objective (NA 1.4). The fluorescence intensity profile of DrM1 and DrM61 spastin in SMN axons (*n*=6 and *n*=8, respectively) was estimated along a 25 μm long lane starting from the distal tip of the axon (ImageJ). The fluorescence intensity background was measured in an adjacent region of the axon using the same region of interest and was subtracted from each spastin value before statistical analysis.

### *In vivo* time-lapse videomicroscopy

MO*sp^ATG1^*, MO*sp^ATG2^* and MOscr-injected Tg(*Hb9*:GFP) transgenic embryos were anesthetised at 40 hpf with tricaine and embedded in 0.8% low melting agarose in a 35 mm glass dish (Iwaki). Time-lapse videomicroscopy recordings of sMN axon navigation were carried out using a Leica DMI 6000B inverted spinning-disk microscope with a 40×/1.25 NA immersion objective over a period of 32 h. *Z*-stacks of 1 µm were taken over a 70 µm depth every 8 min and compiled into time-lapse movies or figure panels.

### Cell culture, transfection and immunocytochemistry

COS-7 (ATCC CRL1651) cells were cultured in DMEM supplemented with 10% foetal calf serum at 37°C under 5% CO_2_. cDNA constructs were transfected using Lipofectamine 2000 according to the manufacturer's instructions (Life Technologies) with a DNA:Lipofectamine ratio of 1:1.5. Cells were fixed 24 h post-transfection in a 4% PFA/4% sucrose solution for 20 min, blocked in 3% PBS-BSA supplemented with 5% normal goat serum and incubated with HA (1:100, 11867423001, Roche) and tyrosinated tubulin (1:4000, T9028, Sigma) antibodies. After several washes in PBS, cells were incubated with appropriate Alexa Fluor-conjugated secondary antibodies.

### Western blot analysis

COS-7 cells and zebrafish embryos were respectively lysed in SDS-lysis buffer [25 mM sodium phosphate (pH 7.2), 5 mM EDTA, 1% SDS] and SDS sample buffer [0.5 µl per embryo; 1 M Tris-HCl (pH 6.8)/10% glycerol/5% β-mercaptoethanol/3.5% SDS], supplemented with a cocktail of protease inhibitors (Roche). A total of 10 µg of zebrafish protein extracts and 5 µg of total protein lysates from COS-7 mock and transfected cells were electrophoresed into 10% SDS-PAGE gel and transferred onto nitrocellulose membranes. Immunoblotting was performed after overnight incubation at 4°C with HA (1:5000, 11867423001, Roche), spastin86-340 (1:1000; Connell et al., 2009), H2b (1:16,000, ab1790, Abcam) and actin (1:10,000, AC-40, Sigma) antibodies. Immunostained proteins were visualised using appropriate peroxydase-labelled antibodies (Jackson ImmunoResearch) and a chemiluminescence detection system (Santa Cruz Biotechnology). DrM1 and DrM61 levels were estimated by quantifying blot band density normalised to H2b values (ImageJ software).

### Statistics

All data were obtained from at least three independent experiments and are presented as mean±s.e.m. The statistical significance of the data was evaluated using the non-parametric Mann–Whitney test when comparing two groups (assuming non-Gaussian distribution) or using the Kruskal–Wallis ANOVA test with Dunn's post test or the one-way ANOVA test with Bonferroni's post-test when analysing more than two groups (assuming non-Gaussian distribution or Gaussian distribution, respectively). Data distribution was tested for normality using the D'Agostino and Pearson omnibus normality test. For rescue experiments of the *Nrp1* morphant phenotype, the statistical significance in the distribution of the morphological phenotypes observed in the different experimental conditions was evaluated using the χ^2^ test. All statistical analyses were carried out using GraphPad Prism. *P*-values less than 0.05 were considered significant.

## Supplementary Material

Supplementary information
