# Supplementary material for: BMP- and neuropilin 1-mediated motor axon navigation relies on spastin alternative translation
Source: Development. 2018 Sep 12;145(17):dev162701. doi: 10.1242/dev.162701 (PMC6141775; doi:10.1242/dev.162701)
Supplement: Supplementary information [file develop-145-162701-s1.pdf]

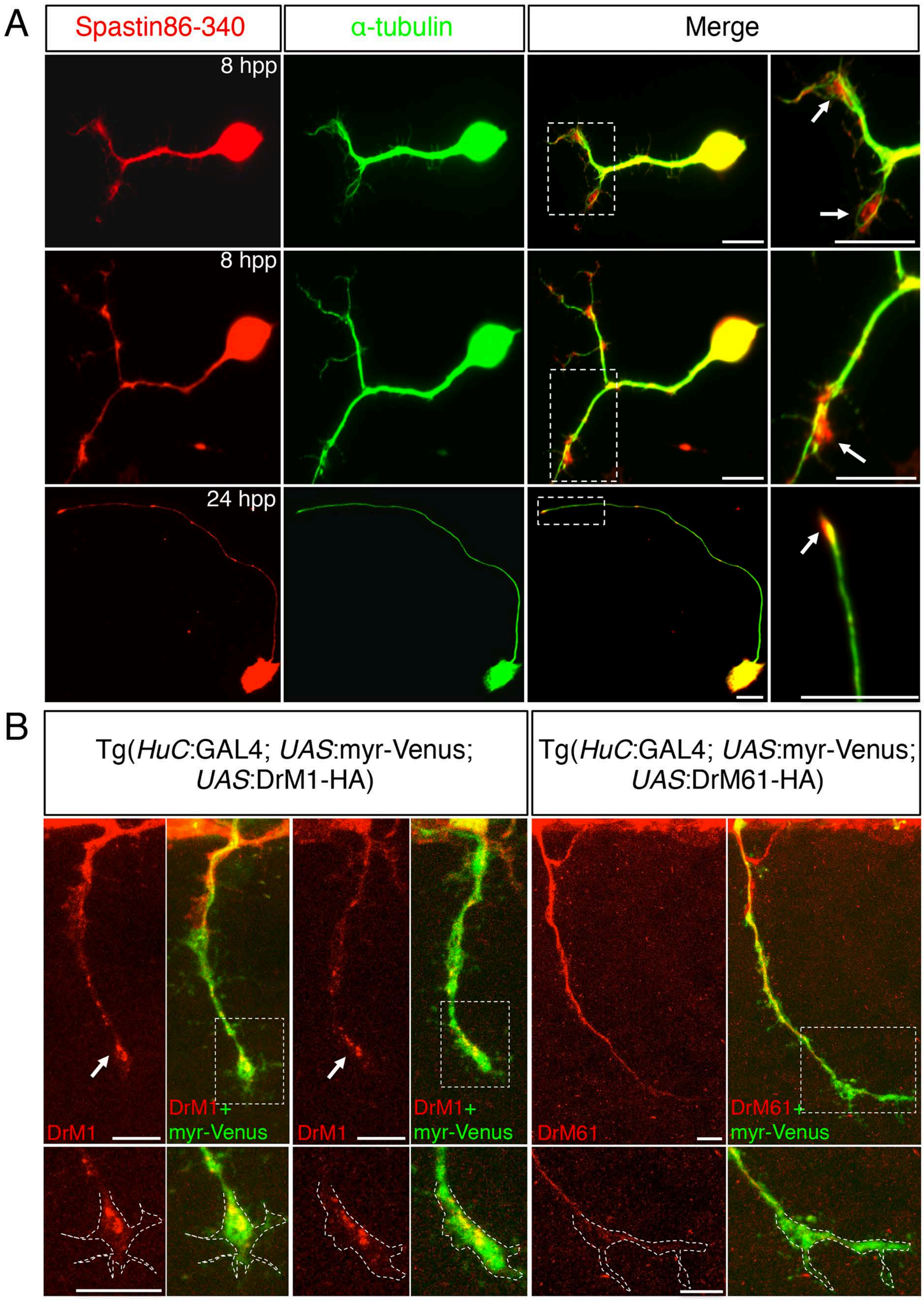

Fig. S1

Development • Supplementary information

Figure S1. Subcellular distribution of DrM1 and DrM61 spastin isoforms in zebrafish spinal neurons. (A) Primary cultures of zebrafish spinal neurons were immunolabelled 8 or 24 hour post-plating (hpp) with spastin 86-340 (red) and α-tubulin (green) antibodies. Arrows indicate spastin enrichment in growth cones. Right panels are higher magnifications of the boxed region in the corresponding left panel. (B) Tg (*HuC*:Gal4; *UAS*:myr-Venus; *UAS*:DrM1) and Tg (*HuC*:Gal4; *UAS*:myr-Venus; *UAS*:DrM61) embryos were immunolabelled at 28-hpf with HA and GFP antibodies. Arrows point at DrM1 spastin enrichment in SMN growth cones. Bottom panels represent higher magnifications of the boxed region in the corresponding upper panel. Dashed lines in bottom panels delineate the growth cone area. (A-B) Scale bars: 10 μm.

Development • Supplementary information

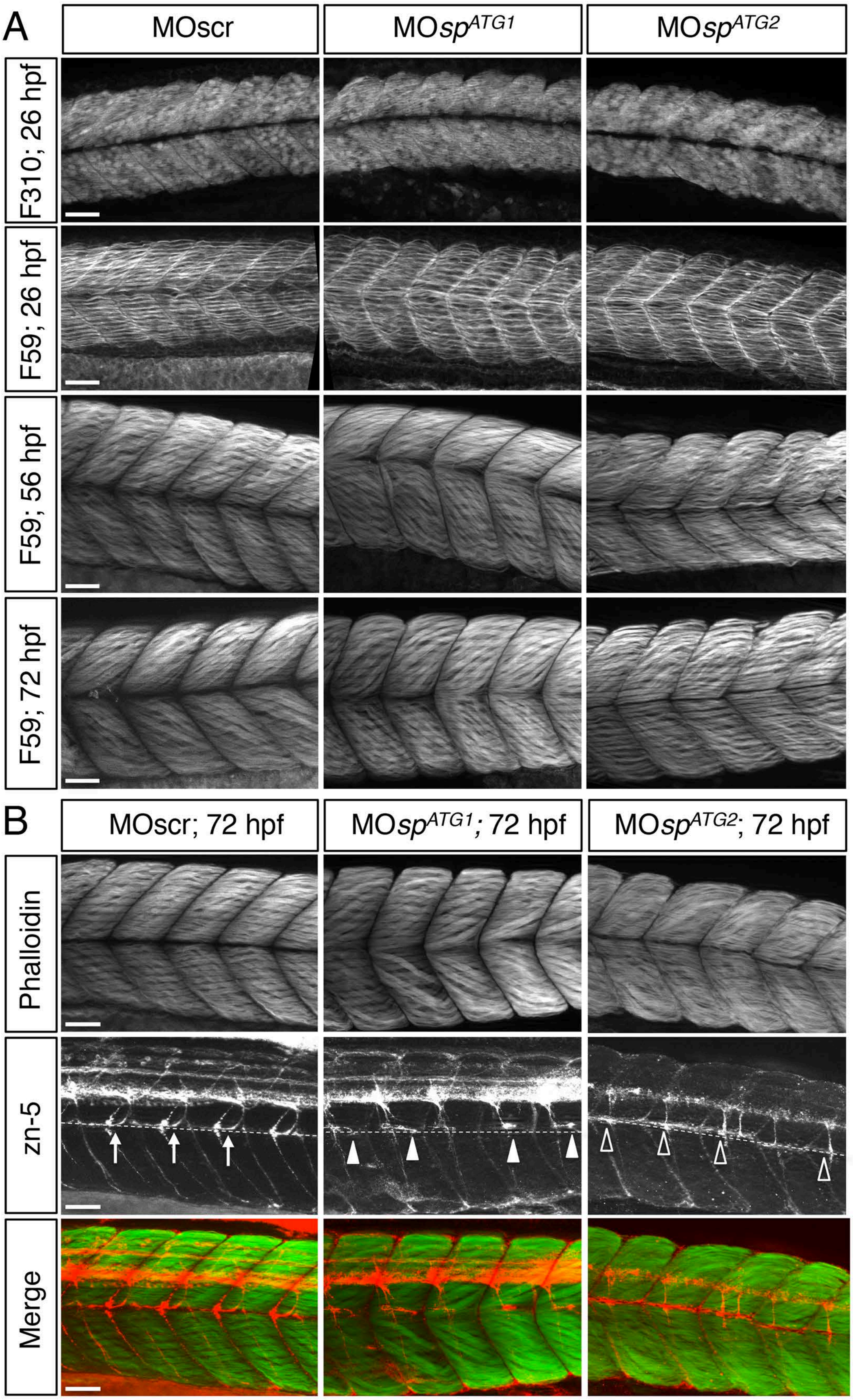
A
MOscr
MOsp^ATG1
MOsp^ATG2
F310; 26 hpf
F59; 26 hpf
F59; 56 hpf
F59; 72 hpf
B
MOscr; 72 hpf
MOsp^ATG1; 72 hpf
MOsp^ATG2; 72 hpf
Phalloidin
zn-5
Merge

Fig. S2

Figure S2. Axon pathfinding defects of ATG1 and ATG2 morphants are not associated with obvious alterations of muscle fibres. (**A**) Immunolabelling of fast (F310 antibody) and/or slow (F59 antibody) muscle fibres in 26-, 56- or 72-hpf embryos injected with MOscr, MO*sp*^{ATG1} or MO*sp*^{ATG2} morpholinos. Slow and fast muscle fibres are well specified and develop properly in morphant embryos or larvae compared to controls. ATG2 morphants only show a slight developmental delay. (**B**) Co-immunolabelling of sMN axons (zn-5 antibody) and muscle fibres (phalloïdin) in 72-hpf control, ATG1 and ATG2 morphant larvae. Dotted lines indicate the horizontal myoseptum. Arrows point at control rostral nerves, while full arrowheads and empty arrowheads respectively indicate misguided or missing rostral nerves in ATG1 or ATG2 morphants. Secondary motor axons of ATG1 and ATG2 morphant larvae fail to navigate properly in an apparently normal muscular environment. (A-B) Scale bars: 50 µm.

Development • Supplementary information

Supplementary information

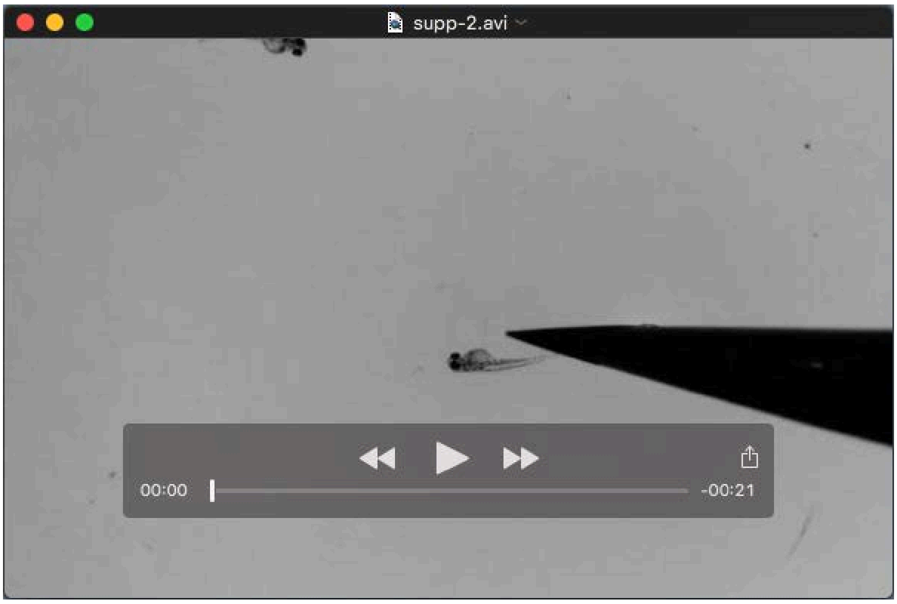

Movie 1. ATG1 and ATG2 morphants show obvious and different locomotor deficits.

Monitoring of touch-evoked escape behaviours of 72-hpf larvae injected with MO*sp*^{ATG1}.

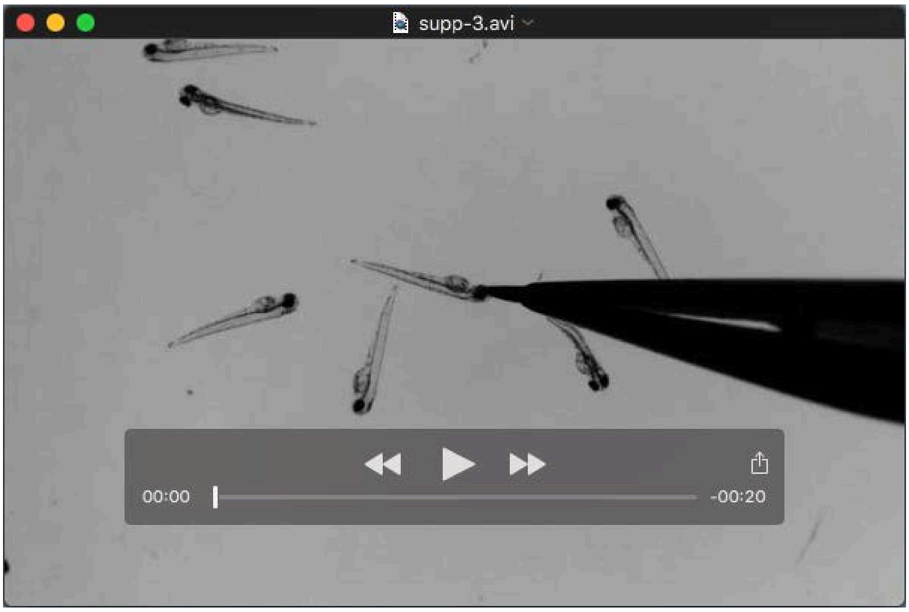

Movie 2. ATG1 and ATG2 morphants show obvious and different locomotor deficits.

Monitoring of touch-evoked escape behaviours of 72-hpf larvae injected with COMO*sp*^{ATG1} control morpholino.

Development • Supplementary information

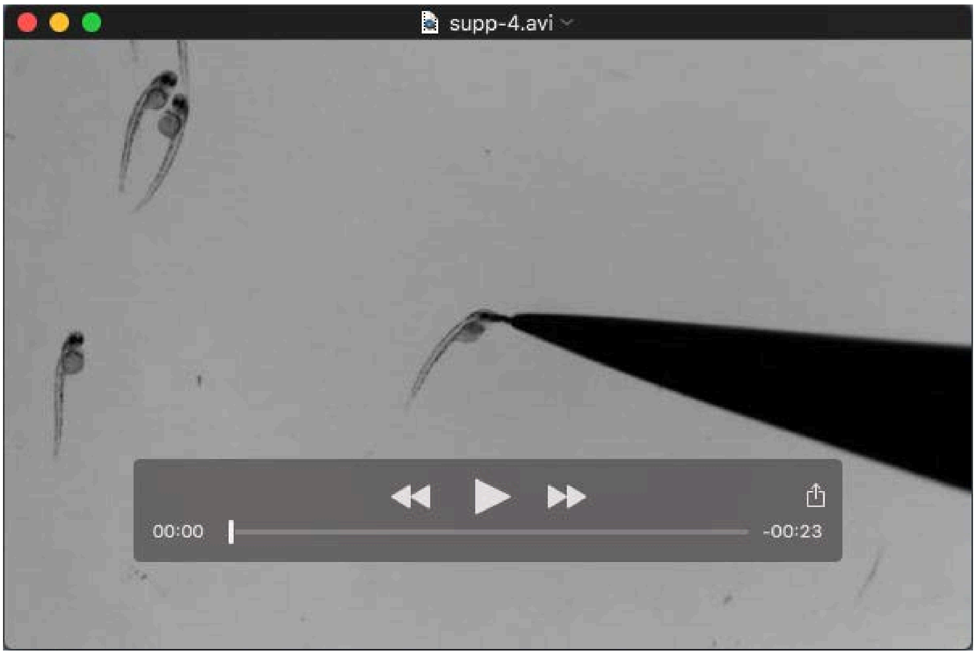

Movie 3. ATG1 and ATG2 morphants show obvious and different locomotor deficits. Monitoring of touch-evoked escape behaviours of 72-hpf larvae injected with MO*sp*^{ATG2}.

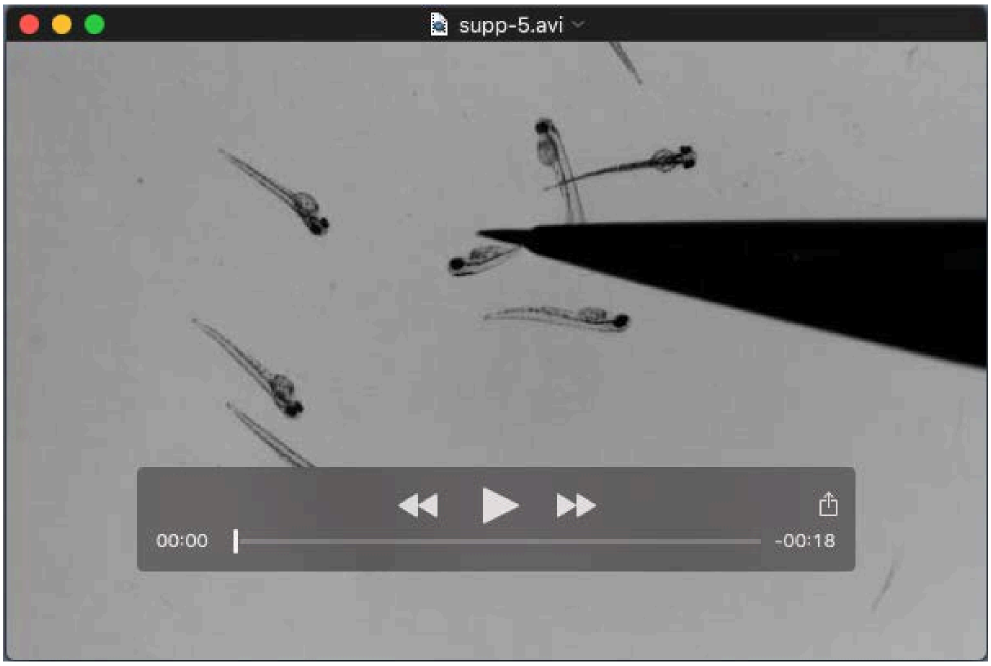

Movie 4. ATG1 and ATG2 morphants show obvious and different locomotor deficits. Monitoring of touch-evoked escape behaviours of 72-hpf larvae injected with COMO*sp*^{ATG2} control morpholino.

Development • Supplementary information

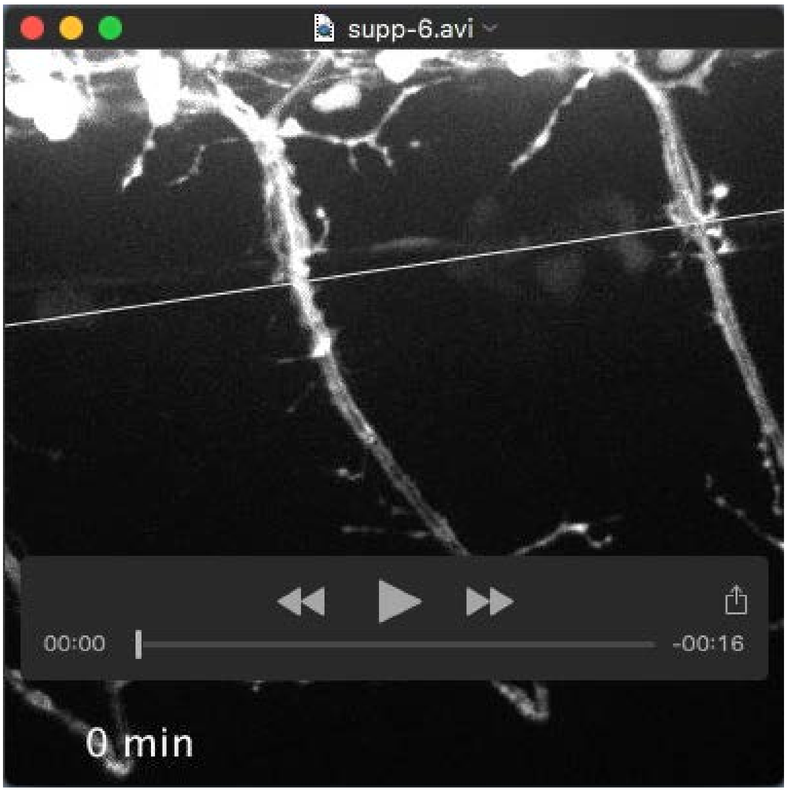

Movie 5. Distinctive navigational behaviour of ATG1 and ATG2 morphant sMN axons at a guidance choice point. Time-lapse recordings of sMN axon outgrowth in 40-72-hpf Tg(*Hb9*:GFP) transgenic larvae injected with MOscr control morpholino. The white line indicates the horizontal myoseptum. Images were acquired every 8 minutes with a spinning disk microscope.

Development • Supplementary information

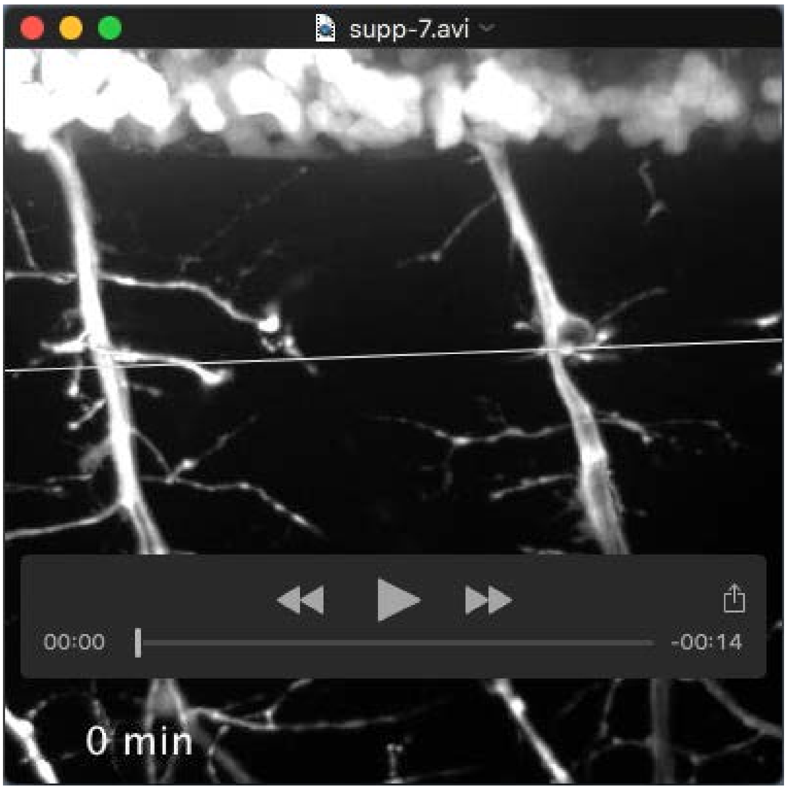

Movie 6. Distinctive navigational behaviour of ATG1 and ATG2 morphant sMN axons at a guidance choice point. Time-lapse recordings of sMN axon outgrowth in 40-72-hpf Tg(*Hb9*:GFP) transgenic larvae injected with MO*sp*^{ATG1} morpholino. The white line indicates the horizontal myoseptum. Images were acquired every 8 minutes with a spinning disk microscope.

Development • Supplementary information

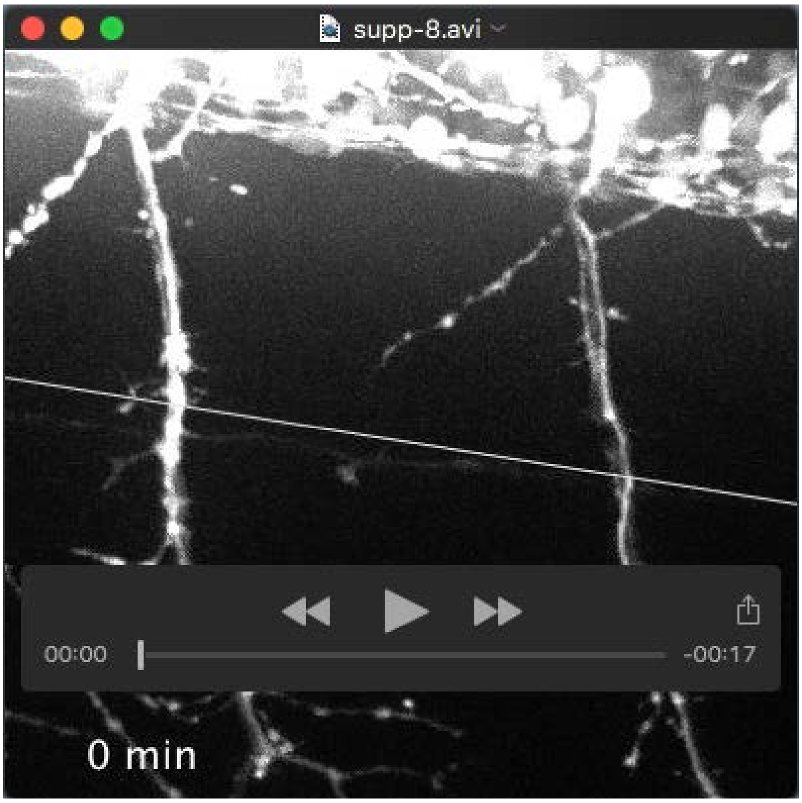

Movie 7. Distinctive navigational behaviour of ATG1 and ATG2 morphant sMN axons at a guidance choice point. Time-lapse recordings of sMN axon outgrowth in 40-72-hpf Tg(*Hb9*:GFP) transgenic larvae injected with MO*sp*^{ATG2} morpholino. The white line indicates the horizontal myoseptum. Images were acquired every 8 minutes with a spinning disk microscope.

Development • Supplementary information

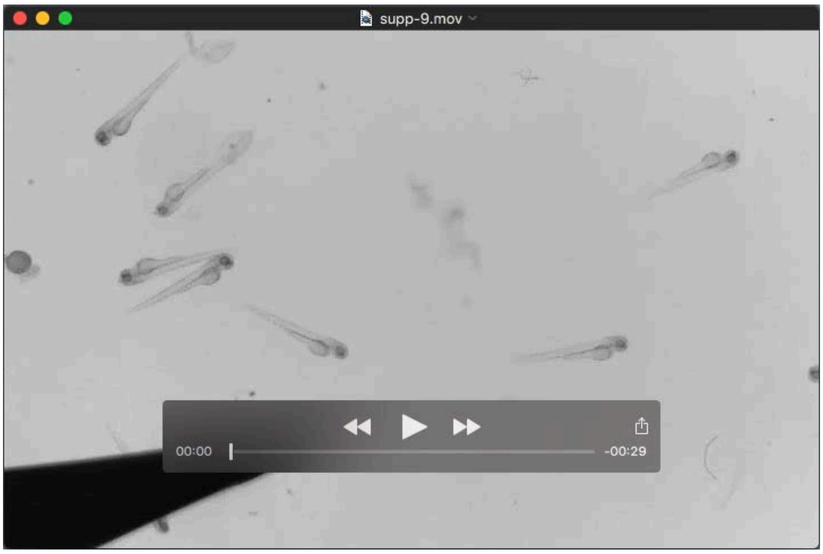

Movie 8. CRISPR/Cas 9 spastin (*sp*^{C68X}) mutant shows locomotor deficits in the touch-escape response test. Monitoring of touch-evoked escape behaviours of 72-hpf larvae obtained from incross among control (*sp*$^{+/+}$) fish.

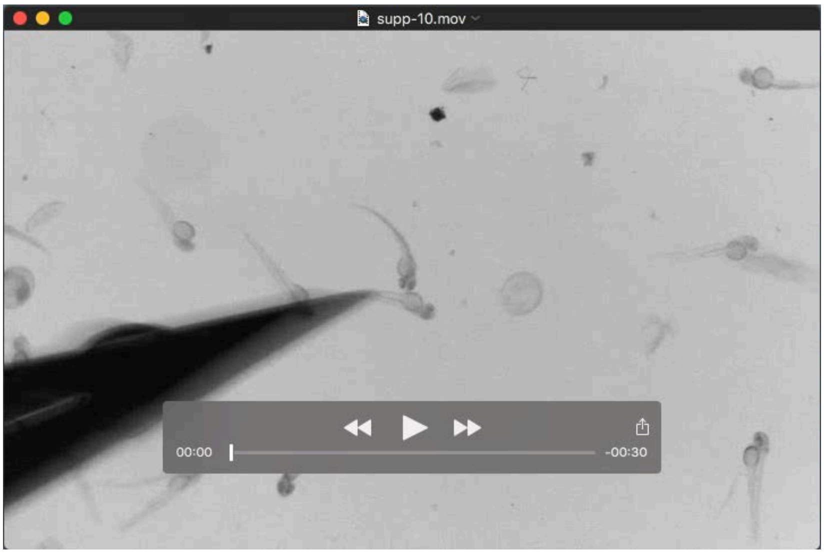

Movie 9. CRISPR/Cas 9 spastin (*sp*^{C68X}) mutant shows locomotor deficits in the touch-escape response test. Monitoring of touch-evoked escape behaviours of 72-hpf larvae obtained from crosses between *sp*$^{C68X/+}$ and *sp*$^{C68X/C68X}$ fish.

Development • Supplementary information

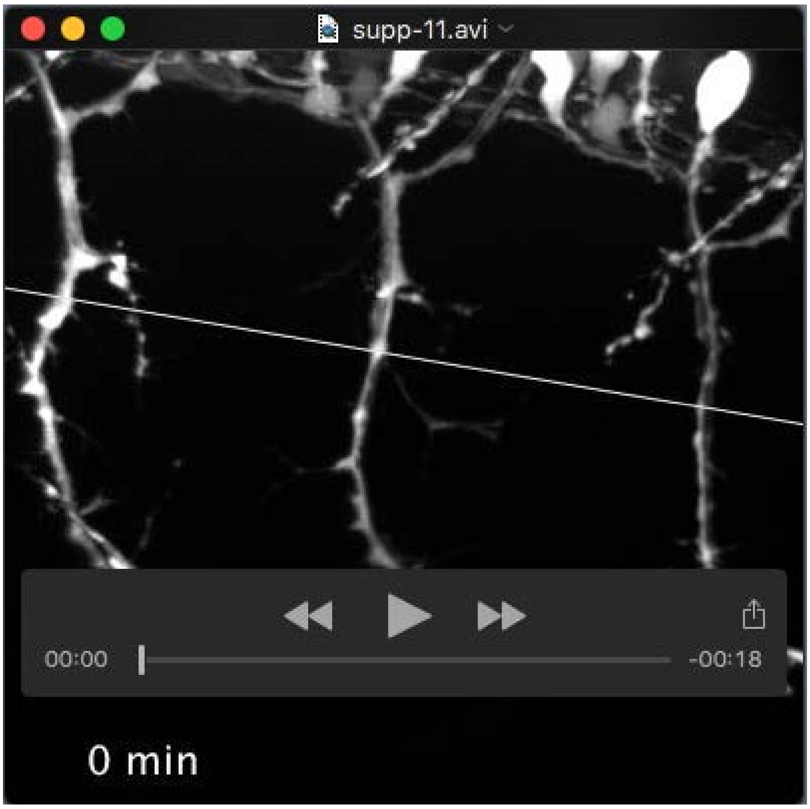

Movie 10. Navigational behaviour of *nrp1a* morphant SMN. Time-lapse recording of sMN development in 40-72-hpf Tg(*Hb9*:GFP) transgenic larvae injected with MO*nrp1a* using a spinning disk microscope. The white line indicates the horizontal myoseptum. Frames were taken every 8 minutes.
